# A gene dosage‐dependent effect unveils NBS1 as both a haploinsufficient tumour suppressor and an essential gene for SHH‐medulloblastoma

**DOI:** 10.1111/nan.12837

**Published:** 2022-08-10

**Authors:** Marialaura Petroni, Francesca Fabretti, Stefano Di Giulio, Vittoria Nicolis di Robilant, Veronica La Monica, Marta Moretti, Francesca Belardinilli, Francesca Bufalieri, Anna Coppa, Paola Paci, Alessandro Corsi, Enrico De Smaele, Sonia Coni, Gianluca Canettieri, Lucia Di Marcotullio, Zhao‐Qi Wang, Giuseppe Giannini

**Affiliations:** ^1^ Department of Molecular Medicine University La Sapienza Rome Italy; ^2^ Department of Experimental Medicine University La Sapienza Rome Italy; ^3^ Department of Computer Engineering, Automation and Management University La Sapienza Rome Italy; ^4^ Institute for Systems Analysis and Computer Science Antonio Ruberti National Research Council Rome Italy; ^5^ Leibniz Institute on Aging‐Fritz Lipmann Institute (FLI) Jena Germany; ^6^ Istituto Pasteur‐Fondazione Cenci Bolognetti Rome Italy

**Keywords:** haploinsufficiency, MRN complex, Nijmegen breakage syndrome, Notch, Sonic Hedgehog

## Abstract

**Aims:**

Inherited or somatic mutations in the *MRE11*, *RAD50* and *NBN* genes increase the incidence of tumours, including medulloblastoma (MB). On the other hand, MRE11, RAD50 and NBS1 protein components of the MRN complex are often overexpressed and sometimes essential in cancer. In order to solve the apparent conundrum about the oncosuppressive or oncopromoting role of the MRN complex, we explored the functions of NBS1 in an MB‐prone animal model.

**Materials and methods:**

We generated and analysed the monoallelic or biallelic deletion of the *Nbn* gene in the context of the SmoA1 transgenic mouse, a Sonic Hedgehog (SHH)‐dependent MB‐prone animal model. We used normal and tumour tissues from these animal models, primary granule cell progenitors (GCPs) from genetically modified animals and NBS1‐depleted primary MB cells, to uncover the effects of NBS1 depletion by RNA‐Seq, by biochemical characterisation of the SHH pathway and the DNA damage response (DDR) as well as on the growth and clonogenic properties of GCPs.

**Results:**

We found that monoallelic *Nbn* deletion increases SmoA1‐dependent MB incidence. In addition to a defective DDR, *Nbn* ^+/−^ GCPs show increased clonogenicity compared to *Nbn* ^+/+^ GCPs, dependent on an enhanced Notch signalling. In contrast, full *Nbn* ^KO^ impairs MB development both in SmoA1 mice and in an SHH‐driven tumour allograft.

**Conclusions:**

Our study indicates that *Nbn* is haploinsufficient for SHH‐MB development whereas full *Nbn* ^KO^ is epistatic on SHH‐driven MB development, thus revealing a gene dosage‐dependent effect of *Nbn* inactivation on SHH‐MB development.

## Graphical abstract

The dual role of Nbs1 in Sonic Hedgehog (SHH)‐medulloblastoma (MB). In cancer prone SmoA1‐Granule Cell Progenitors (GCPs), *Nbn* ^KO^ causes reduced proliferation and increased cell death, ultimately leading to inhibition of MB development, due to activation of the DDR and impairment of the SHH pathway. On the other hand, *Nbn* hemizygosis (*Nbn* ^HZ^) causes DDR activation and Notch‐dependent increase in clonogenic capability, favoring GCP transformation. These results, collectively, indicate a gene dosage‐dependent function of Nbn in SHH‐driven tumorigenesis.

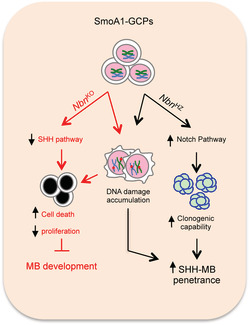

**Key points**

- Monoallelic *Nbn* deletion promotes SHH‐MB in the SmoA1 mouse model.
- Monoallelic *Nbn* deletion causes defective DDR in GCPs.
- *Nbn* ^+/−^ GCP features enhanced Notch‐dependent clonogenicity.
- Full *Nbn* ^KO^ impairs SHH‐driven MB in the SmoA1 model and in tumour allografts.

## INTRODUCTION

Medulloblastoma (MB) is one of the most common and frequently lethal childhood malignant brain tumours. Four consensus molecular groups are currently defined by distinct multiomic patterns and clinical features [1]. Groups 1 and 2 are typically linked to genetic aberrations and deregulated gene expression patterns of the homologous Wingless and Int‐1 (WNT) and Sonic Hedgehog (SHH) pathways, respectively. Groups 3‐MB and 4‐MB present more heterogeneous molecular features, sharing relatively frequent alterations of Chromosome 17 and an enrichment for amplification of MYC genes. Based on the more extensive and integrated use of multiomic approaches, histological reports and clinical data, each of these core groups may be further divided into additional subgroups [2, 3, 4].

Mutations in critical regulators of the WNT (e.g., *APC*) and SHH (e.g., *Ptch1*, *SUFU* and *Smo*) pathways confer increased risk for MB development [5]. In particular, WNT‐MBs develop from progenitor cells of the lower rhombic lip, whereas SHH‐MBs arise from the granule cell progenitors (GCPs) in the external germinal layer of the developing cerebellum. Intriguingly, the DNA damage/repair response (DDR) pathway also plays an important role in MB development. For example, mutations in *BRCA2*, *PALB2* and *TP53* confer an increased risk of MB, most often of the SHH type [5]. Studies in animal models complemented these observations by showing that inactivation of Non‐Homologous End‐Joining (NHEJ) and Homologous Recombination (HR) genes, such as *Lig4*, *Xrcc4*, *Ku80*, *Xrcc2*, *Brca2* and *Parp1*, leads to MB development in p53‐deficient backgrounds [6, 7, 8, 9, 10].

The MRE11/RAD50/NBS1 (MRN) complex is a crucial orchestrator of the DDR [11], disruption of which results in embryonic lethality in mice [12, 13, 14]. In humans, homozygous hypomorphic mutations in *MRE11*, *NBN* (coding for the NBS1 protein) and *RAD50* genes cause ataxia telangiectasia‐like disorder (ATLD‐OMIM:604391), Nijmegen breakage syndrome (NBS‐OMIM:251260) and NBS‐like disorder (NBSLD‐OMIM:613078), respectively. These are typically associated with genome instability, immunodeficiency and developmental defects of neural tissues [15]. The Nbn‐CNS‐del mouse, which carries full *Nbn* knockout restricted to the central nervous system (CNS), recapitulates some of the neural phenotypes of the above‐mentioned syndromes such as microcephaly and dramatic cerebellar atrophy associated with ataxia [16, 17], indicating that the MRN complex is essential for cerebellar development and for the expansion of cerebellar GCPs [16, 18].

Notably, the role of the MRN complex in cancer is multifaceted. On one side, MRN‐associated syndromes are characterised by cancer predisposition [15] (http://www.cancerindex.org/Nijmegen_Breakage_Syndrome#section9), highlighting an oncosuppressive function for this complex. This is also supported by mouse models [14, 19, 20]. In particular, monoallelic *Nbn* knockout significantly increased the occurrence of lymphoma and solid tumours on its own or after γ‐ray exposure, supporting the hypothesis that *Nbn* is haploinsufficient for cancer suppression [21, 22]. Monoallelic variants of the MRN genes have been reported in many human tumours (reviewed in [23, 24]). With respect to MB, not only has it been sporadically described in NBS patients [25, 26], but heterozygous germline or somatic *NBN*, *RAD50* and *MRE11* mutations were discovered in MB patients, implying their potential role as haploinsufficient tumour suppressors for MB development [27, 28, 29, 30].

On the other hand, the MRN complex also exerts oncopromoting functions and confers survival advantages to tumour cells by facilitating resistance to oncogene‐induced RS, as demonstrated for MYCN‐driven neuroblastoma [31] and c‐MYC‐driven B‐cell lymphomas [32]. Increased expression of MRN genes has been observed in MYCN‐driven tumours and other cancer types, including prostate, gastric, rectal and head‐and‐neck cancers [31, 33].

The apparent conundrum generated by the oncosuppressive and oncopromoting functions of the MRN complex has not yet been clearly reconciled. Here, we report that monoallelic or full *Nbn* deletion in an SHH‐MB‐prone mouse model supports a role of this gene as a haploinsufficient oncosuppressor or an oncopromoting factor, respectively, providing evidence for a gene/dosage‐dependent effect on MB development.

## MATERIALS AND METHODS

### Mouse models and genotyping

The ND2:SmoA1 mice [34] and the *Nbn*‐CNS‐del mice [16] were previously described. By crossing ND2:SmoA1 transgenic mice, *Nbn* ^F6/F6^ mice and nestin‐Cre mice, we obtained ND2:SmoA1/*Nbn* ^F6/F6^;Cre^+^ mice (here called SmoA1/*Nbn* ^KO^) and ND2:SmoA1/*Nbn* ^+/F6^;Cre^+^ mice (here called SmoA1/*Nbn* ^HZ^). The SmoA1 transgene copy number was evaluated via qPCR analysis.

Gli1^tm3(Cre/ERT2)Alj^ [35] (here called Gli1CreER) mice were purchased from Jackson Laboratories and crossed with *Nbn* ^F6/F6^ mice to obtain *Nbn* ^+/F6^/Gli1CreER^+/−^ mice. All breeding strategies were designed in order to compare mice with the same genetic background.

Genomic DNA was isolated from tail biopsies. Methods, primers and probe sequences for genotyping are given in Tables S1 and S2.

For mouse allografts, MB neurospheres (500,000 cells per flank) were suspended in an equal volume of medium and Matrigel (BD Biosciences, Heidelberg, Germany) and injected into the posterior flank of female BALB/c nude mice (Nu/Nu) (Charles River Laboratories, Lecco, Italy). When measurable, tumour size was calculated by the formula length × width × 0.5 × (length + width). For survival studies, mice were sacrificed when the tumour size reached 2 cm^3^. Mice not meeting these criteria earlier were sacrificed 66 days from the beginning of tumour size measurement, at the latest.

### GCP cultures, SAG‐dependent neurospheres and MB neurospheres

GCPs were isolated from P5 or P7 mouse cerebella and grown as standard cultures or Smo agonist (SAG)‐dependent cerebellar neurospheres (S‐cNS) as described in [18, 36], respectively. SAG (AG‐CR1‐3506, AdipoGen Life Sciences, San Diego, CA, USA) was added to GCP neurospheres at a 200 nM concentration unless otherwise specified. MB neurospheres were prepared and grown as described [37]. Whenever necessary, GCP and MB neurospheres were pelleted and dissociated by incubation with Accutase (Gibco) to obtain single‐cell suspensions.

For neurosphere‐forming assays, dissociated cells were seeded at 20 cells/well in 96 well plates in the appropriate medium with SAG and with or without *mirin* (40 μM, Sigma Aldrich), GDC‐0449 (0.5 μM, S1082, Selleckchem, Houston, TX, USA) or DAPT (10 μM, 565,770, Merk Millipore, Darmstadt, Germany). Thirty technical replicates were performed for each experiment. The number of spheres/well was counted after 2 weeks.

In order to induce *Nbn* monoallelic KO, *Nbn* ^+/−^/Gli1CreER^+/−^ neurospheres were dissociated and seeded at 1x10^5^ cells/cm^2^ in poly‐L‐Lysine (40 μg/ml) coated dishes with the adhesion medium (details in Supplementary Information). After three days, subconfluent adhering cells were treated with 1 μM 4‐hydroxytamoxifen (SC‐3542, Santa Cruz Biotechnology) or ethanol for 24 h and analysed 48 h after tamoxifen wash out.

For *Nbn* RNA interference, MB neurospheres were dissociated and electroporated with 100 nM of anti‐*Nbn* Stealth RNAi (set of 3: MSS219515, MSS219516, MSS219517, #1320001, Invitrogen) or negative control Stealth RNAi (#452001, Invitrogen) by the Amaxa Mouse NSC Nucleofector kit (VPG‐1004, Lonza, Basel Switzerland) in a Nuclefector II (Amaxa Biosystems, Gaithersburg, MD, USA).

### Immunohistochemistry and immunofluorescence assays

Formalin‐fixed and paraffin‐embedded (FFPE) tissue sections were processed for H&E staining. Images were acquired using a Leica DM1000 microscope.

For EdU incorporation assay, cells were incubated with EdU (10 μM) for 1 h (S‐cNS cultures) or 24 h (GCPs standard cultures) and fixed with 3.7% formaldehyde in PBS. EdU incorporation was evaluated using the Click‐iT™ EdU Cell Proliferation Kit (Thermo Fisher Scientific), according to the manufacturer's instructions. Live/dead cell analysis was performed using the Live/Dead Cell Imaging Kit (R37601, Thermo Fisher Scientific), according to the manufacturer's instructions. Fluorescence images were acquired on a LEICA DM 2500 microscope using the IScapture software (Tucsen Photonics Co., Ltd, Fuzhou, Fujian, China). Phase contrast images were acquired on an EVOS XL CORE microscope (Thermo Fisher Scientific).

### RNA and protein extraction, qPCR and Western blot

mRNA and protein extraction were performed as previously described [38]. Quantitative PCR (qPCR) was performed on a ViiA 7 real‐time PCR system (Thermo Fisher Scientific) as previously described [39] or using a custom 384‐Well Microfluidic Card TaqMan Gene Expression Assay (Thermo Fisher Scientific). The list of qPCR assays is given in Supplementary Information. At least three biological replicates were analysed for each experimental condition. All values were normalised on the expression of at least two reference genes. mRNA quantification was quantified through the ΔΔCt method.

Total protein extraction and Western blot protocols were performed as described [38, 40]. Immunoreactive bands were visualised by enhanced chemoluminescence (Advansta Inc., Menlo Park, CA, USA). The list of antibodies is given in Supplementary Table 5.

### Sanger sequencing and RNA sequencing

cDNA was amplified by PCR (list of primers in Supplementary Table 6). Sequencing was performed using the BigDye Terminator v3.1 Cycle Sequencing Kit and a 3130XL Genetic Analyser (Applied Biosystems).

RNA sequencing in service (Genomix4Life S.r.l., Baronissi [SA] Italy) was performed by Illumina NextSeq and sequenced in paired‐end mode. For each experimental condition, three biological replicates were analysed. The method for quantification of transcripts is given as Supplementary Information. Heatmap of expression values for transcripts (rows) across samples (columns) was generated by using the pheatmap R package. Gene expression values are z‐score normalised across rows and expression levels increase from blue (low) to red (high). Scatter plots are generated in R by using the library *plotly* on the mean of the expression levels (in log‐scale) across samples.

### Public dataset

R2‐Genomics analysis and visualisation platform (http://r2.amc.nl) were used to investigate the expression of *NBN*, *MRE11* and *RAD50* genes in MB patients. MB dataset: (SHH)‐Pfister‐73‐MAS5.0, GSE49243; normal cerebellum dataset: Roth‐9‐MAS5.0, GSE3526. Data were downloaded from the website and formatted for publication.

### Statistical analysis

Data are presented as mean from at least three independent experiments ± standard deviation (SD) or ± standard error (SEM), as indicated in the figure legends. Statistical analysis was performed by a standard two‐tailed Student's *t*‐test or ANOVA test, as indicated in the figure legends. Statistical significance of the differences in tumour penetrance between *Nbn* ^WT^ and *Nbn* ^HZ^ mice was computed using the one‐tailed chi‐square test. The survival curve was drawn as Kaplan–Meier Cumulative Proportion Surviving graph, and the corresponding *p*‐value was calculated with the Gehan‐Breslow‐Wilcoxon test. Software GraphPRISM6 (La Jolla, CA) was used to calculate the median survival. For all tests, a *p*‐value <0.05 was considered to indicate statistically significant differences.

## RESULTS

### Generation of SmoA1/*Nbn*‐CNS‐del mice

To address the role of NBS1 in MB, we used the ND2:SmoA1 mice in which a transgenic mutant Smo receptor activates the SHH pathway in GCPs, inducing SHH‐dependent MB development [34]. By repeatedly interbreeding these mice, MB penetrance in homozygous ND2:SmoA1 mice (the Smo/Smo model) declined significantly compared to the original model (Figure S1A), as also reported by others [41]. This is possibly due to a counterselection of the mice with a stronger phenotype, rendering the model more suitable for investigating the contribution of MRN dysfunction to MB tumorigenesis. These mice were crossed with *Nbn*‐CNS‐del mice, where *Nbn* is knocked out specifically in the CNS by nestin‐Cre‐dependent excision of its exon 6 [16]. The phenotypes of both ND2:SmoA1/*Nbn* ^+/F6^;Cre^+^ mice (from now on SmoA1/*Nbn* ^HZ^ mice) and ND2:SmoA1/*Nbn* ^F6/F6^;Cre^+^ mice (from now on SmoA1/*Nbn* ^KO^) were studied. *Nbn* gene deletion and impaired protein expression in SmoA1/*Nbn* ^HZ^ and SmoA1/*Nbn* ^KO^ mice, as well as the expression of the SmoA1 transgene, were confirmed as appropriate (Figure S1B, C, D).

### ***Nbn*** **hemizygosity increases** **SmoA1‐dependent MB development**

**FIGURE 1:**
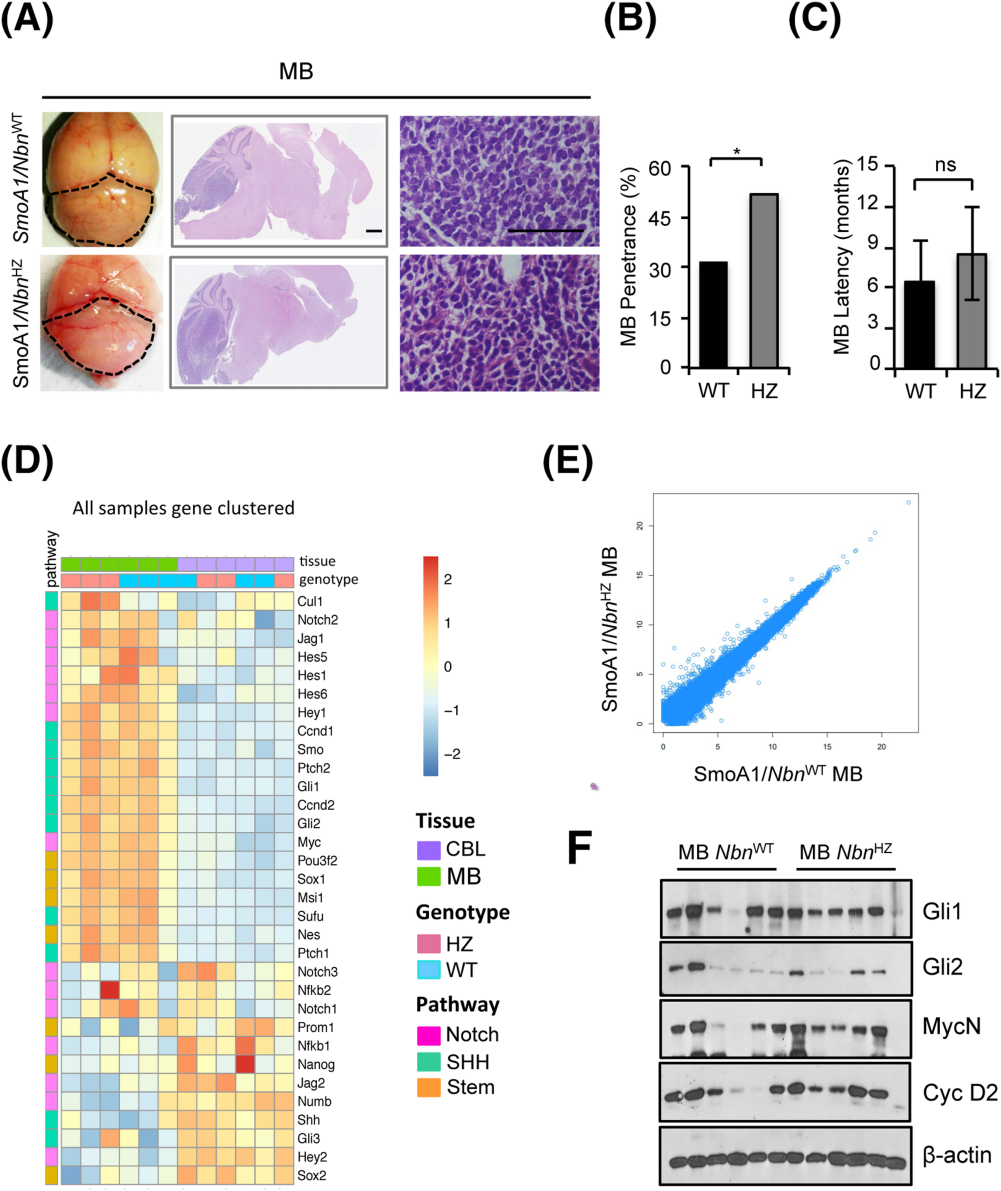
Monoallelic *Nbn* loss enhances Sonic Hedgehog (SHH)‐dependent medulloblastoma (MB) development. (A) Representative images of macroscopic features (left panels), haematoxylin/eosin‐stained sagittal sections (middle panels, scale bar: 1 mm) and high‐magnification view (right panels; scale bar 100 μM) of the brain/cerebella from adult mice with the indicated genotypes. Dotted lines highlight the size/shape of MB‐carrying cerebella. (B and C) Histograms representing MB penetrance (B) and latency (C) in SmoA1/*Nbn* ^WT^ and SmoA1/*Nbn* ^HZ^ mice. *p*‐Values were calculated by a one‐tailed chi‐square test (*p < 0.05) or two‐sided Student's *t*‐test (ns, not significant). (D) Heatmap of the RNA‐Seq data of the expression of the indicated transcripts in MB and healthy adult cerebella (CBL; 15 months) of SmoA1/*Nbn* ^WT^ (WT) and SmoA1/*Nbn* ^HZ^ (HZ) mice. The heatmap colour key denotes the gene expression value increasing from blue (low) to red (high). Gene expression values were clustered according to the column (samples) by exploiting a complete linkage hierarchical clustering algorithm and by using the Euclidean distance as distance metric. Pathway indication for each gene is also reported. (E) Scatter plot of the RNA‐Seq expression data in SmoA1/*Nbn* ^WT^ versus SmoA1/*Nbn* ^HZ^ tumours. Data are reported as the mean of the gene expression levels (in log‐scale) of three independent samples for each genetic background. (F) Western blot (WB) analysis of MB (n = 6) explanted from mice with the indicated genotypes

Hypomorphic and/or monoallelic *MRN* mutations and losses occur in human SHH‐MB[25, 26, 27, 28, 29, 30] (Waszak S. and Pfister S, personal communication). Thus, we examined the effect of monoallelic *Nbn* deletion (*Nbn* ^HZ^) in the SmoA1 background with reduced MB penetrance, to specifically test whether it may facilitate GCP transformation. SmoA1 mice with wild‐type *Nbn* (from now on SmoA1/*Nbn* ^WT^) reached adulthood and as expected, developed clinical signs of MB between 3 and 13 months (Figures 1A,B and S1A). Interestingly, about twice as many SmoA1/*Nbn* ^HZ^ mice, when compared to the SmoA1/*Nbn* ^WT^ ones, showed ataxia, a protruded skull, a tilted head and hunched posture suggesting the presence of a MB, which was further confirmed by macroscopic and histological analyses in all cases (Figure 1A,B). Latency for MB development did not vary significantly between the two genotypes (Figure 1C). Similarly to human MB[27], loss or mutations in the residual *Nbn* allele did not occur in SmoA1/*Nbn* ^HZ^ mice (data not shown).

SmoA1/*Nbn* ^HZ^ and SmoA1/*Nbn* ^WT^ MBs appeared very similar at the histopathological level (Figure 1A). RNA sequencing distinctly showed that all MB samples were different from healthy adult cerebella, showing deregulation of SHH, Notch and stemness pathways (Figures 1D and S2A, B). Even though MBs were correctly clustered for genotype by using a complete linkage hierarchical clustering algorithm (Figure 1D), we found no significant differences between SmoA1/*Nbn* ^HZ^ and SmoA1/*Nbn* ^WT^ MBs (Figure 1E). In particular, analysis of the hedgehog (HH) targets Gli1, MycN and Cyclin D2 proteins confirmed a similar SHH activity in the tumour samples of both backgrounds (Figure 1F).

Overall, these data are consistent with the hypothesis that full *Nbn* gene dosage protects from MB development in SHH‐MB‐prone mice.

### DDR markers do not differ in *Nbn* ^HZ^ and *Nbn* ^WT^ SmoA1‐driven MBs, but can be induced by acute *Nbn* monoallelic deletion in GCP neurospheres

*Nbn* hypomorphic mutations in humans and *Nbn* hemizygosity in mice are associated with genetic instability and increased cancer susceptibility 22, 42, 43], suggesting that *Nbn* might be haploinsufficient in protecting against cancer possibly via impairment of the DDR. However, we failed to detect significant differences in H2AX phosphorylation between SmoA1/*Nbn* ^HZ^ and SmoA1/*Nbn* ^WT^ MBs (Figure S3A) and we did not detect relevant differences in the expression of DDR genes among MBs from the two genotypes (Figure S3A‐F). Rather, we noticed a general upregulation of DNA repair pathways in MB samples compared to healthy cerebella (Figure S3B‐F). Moreover, we found no significant variation in the expression of NBS1 in SmoA1/*Nbn* ^HZ^ versus SmoA1/*Nbn* ^WT^ MBs (Figure S4A and D), overall confirming that full‐blown MB of SmoA1/*Nbn* ^HZ^ and SmoA1/*Nbn* ^WT^ animals do not differ significantly.

In contrast, we observed a significantly lower NBS1 expression in P7 and P14 cerebella from SmoA1/*Nbn* ^HZ^ compared to SmoA1/*Nbn* ^WT^ mice (Figure S4B, C and D), indicating that *Nbn* hemizygosis results in decreased NBS1 expression in developing cerebella and potentially suggesting that an increased compensatory expression by the single *Nbn* allele restores its protein levels in *Nbn* ^HZ^ MBs (Figure S4E).

**FIGURE 2:**
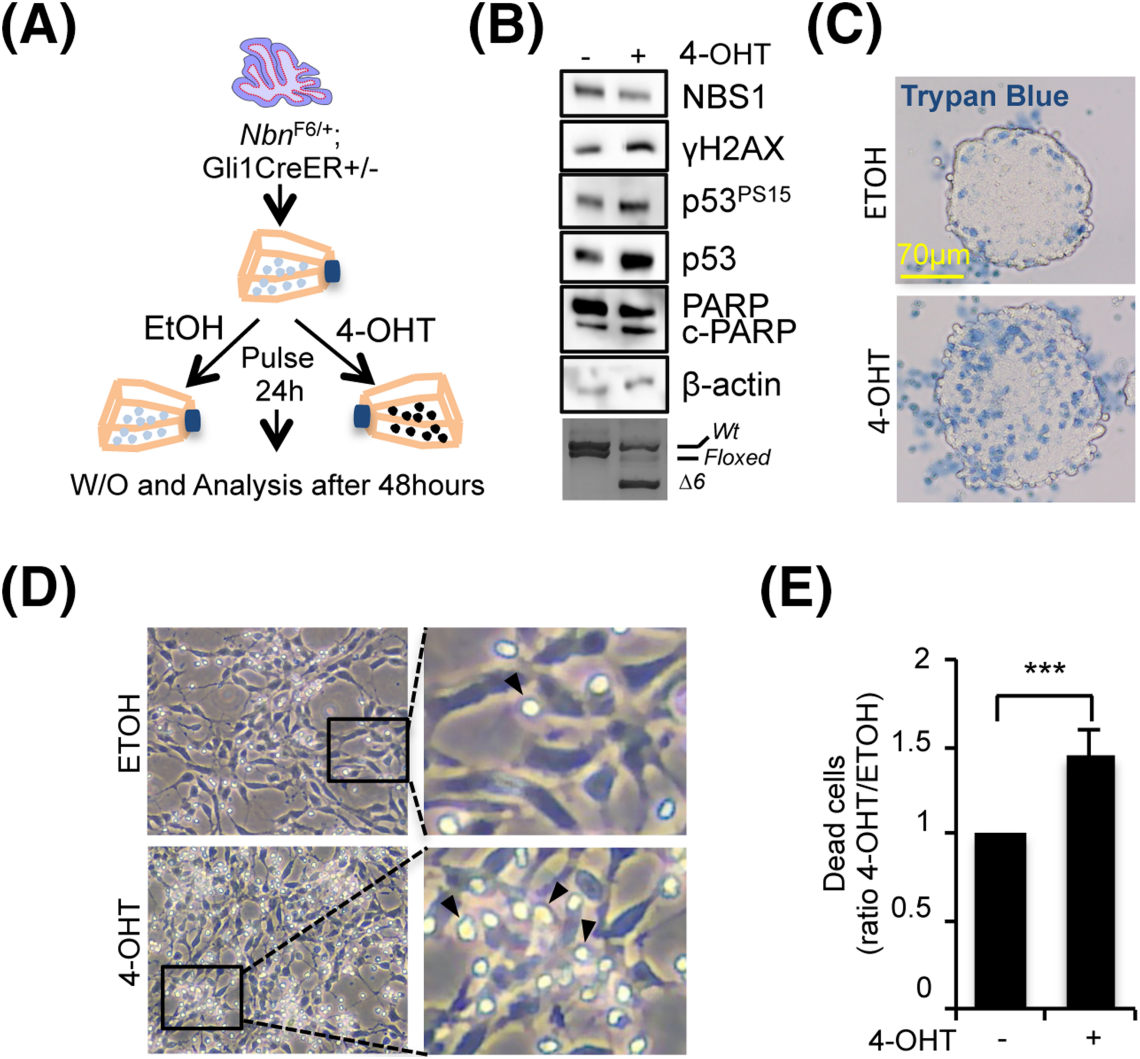
Acute monoallelic *Nbn* loss induces DNA damage response (DDR) activation in Smo agonist (SAG)‐dependent cerebellar neurospheres (S‐cNS). (A) Schematic protocol depicting the generation and manipulation of *Nbn* ^F6/+^;Gli1CreER^+/−^ S‐cNS. (B) Western blot (WB) analysis of the *Nbn* ^F6/+^;Gli1CreER^+/−^ S‐cNS treated with ethanol (ETOH) or 4‐hydroxytamoxifen (4‐OHT) for 24 h and collected 48 h after the washout. Blots were probed with the indicated antibodies, and β‐actin was used as loading control. c‐PARP, cleaved PARP. Cre‐induced *Nbn* gene cleavage identified by PCR is provided in the lowest panel, as an internal control; the size of wild type and floxed alleles are indicated as well as the Δ6 deleted allele. Data are representative of four replicates. (C) Representative images of *Nbn* ^F6/+^;Gli1CreER^+/−^ S‐cNS treated with ETOH or 4‐OHT for 24 h and collected 48 h after the washout. Cells were suspended in a Trypan blue solution to mark dead cells. (D) Representative phase‐contrast images of *Nbn* ^F6/+^;Gli1CreER^+/−^ adherent S‐cNS treated with ETOH or 4‐OHT for 24 h and photographed 48 h after washout. Arrows highlight dead cells (original magnification: 40×). (E) Quantification of cell death in cells treated as in D. Data are representative of three replicates.

To address the consequences of hemizygous *Nbn* ^KO^ and reduced NBS1 expression in the early steps of GCP development, we established primary SAG‐dependent GCP neurospheres (S‐cNS) [36] from P7 *Nbn* ^F6/+^;Gli1CreER^+/−^ mice. In this cellular context, the SHH pathway is constitutively active due to SAG stimulation [36], and Cre‐mediated *Nbn* monoallelic KO can be induced via 4‐hydroxytamoxifen (4‐OHT) administration in vitro (Figures 2A,B and S5A). Acute *Nbn* monoallelic KO reduced NBS1 expression in 4‐OHT‐treated S‐cNS and induced γH2AX, p53^S15P^ and total p53 accumulation (Figure 2B), indicating a DDR activation. Therefore, the higher levels of cleaved PARP (Figure 2B) and induced cell death by 4‐OHT (Figure 2C) are likely due to an excessive accumulation of unrepaired DNA damage. Consistently, the occurrence of cell death was also confirmed in adherent S‐cNS (Figure 2D). Finally, we did not observe similar results in 4‐OHT‐treated S‐cNS from *Nbn* ^F6/+^;Gli1CreER^−/−^mice (Figure S5B), excluding that DDR activation was directly due to 4‐OHT.

Thus, *Nbn* hemizygosis leads to DDR activation in SHH‐driven GCPs.

### ***Nbn*** **hemizygosis** **associates** **with higher clonogenic capability in GCPs**

**FIGURE 3:**
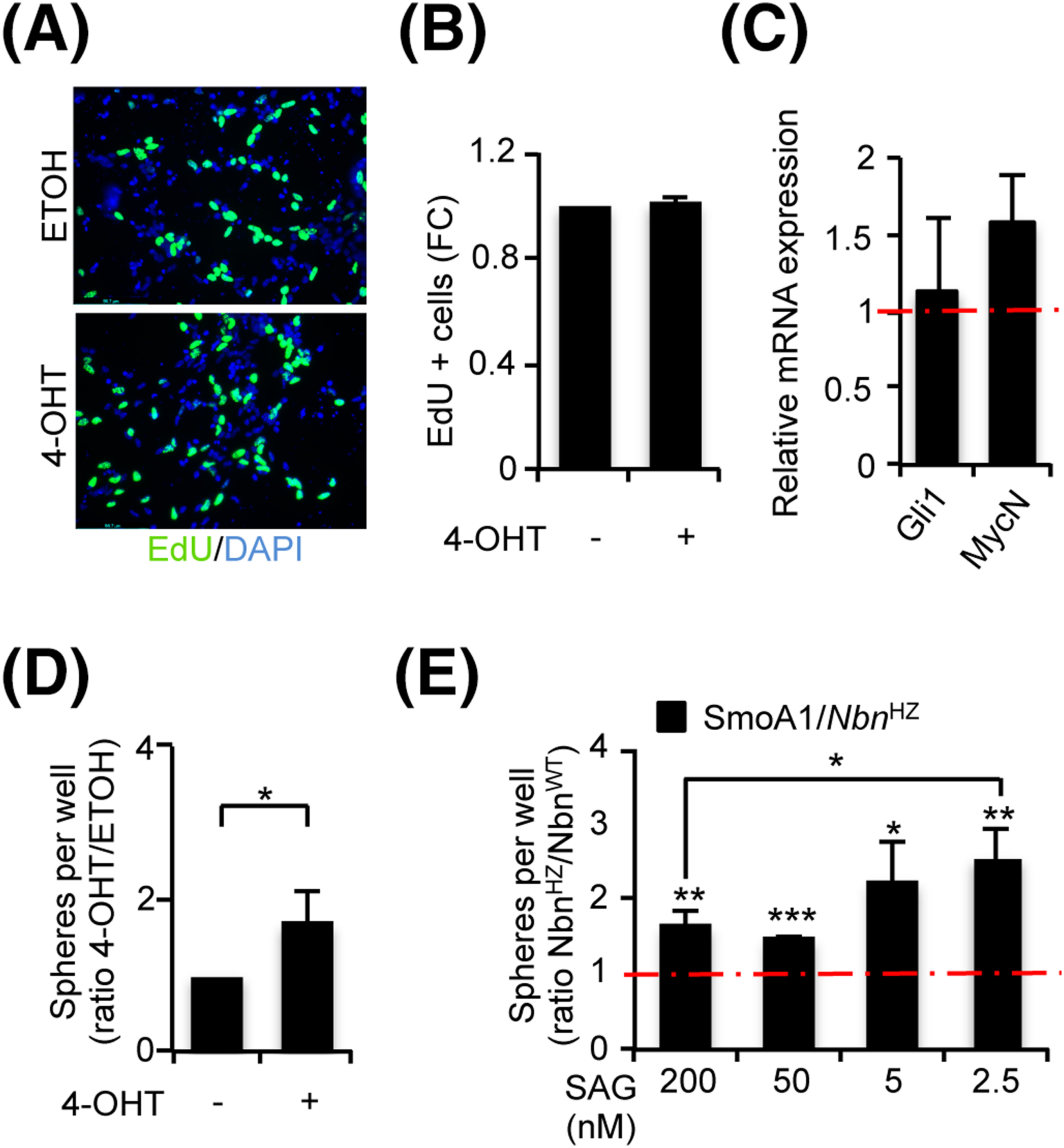
Monoallelic *Nbn* loss enhances clonogenicity in primary granule cell progenitors (GCPs). (A, B) Representative images of EdU staining (A) and graph (B) indicating the percentage of EdU‐positive cells in *Nbn* ^F6/+^;Gli1CreER^+/−^ GCPs treated as in Figure 2C. Data are reported as mean (±SD). (C) qPCR analysis of the indicated transcripts in *Nbn* ^F6/+^;Gli1CreER^+/−^ S‐cNS treated with ethanol (ETOH) or 4‐hydroxytamoxifen (4‐OHT) for 24 h and collected 48 h after washout. mRNA expression levels were normalised on the mean expression of two reference genes (*β2‐microglobulin* and *Hprt*), and relative mRNA quantification was expressed as fold change. Data are reported as mean (±SD). *p*‐Values were calculated by two‐sided Student's *t*‐test (*p < 0.05, **p < 0.01, ***p < 0.001). (D) Neurosphere formation assay from P7 *Nbn* ^F6/+^;Gli1CreER^+/−^ GCPs. Neurospheres were treated with ETOH or 4‐OHT for 24 h, dissociated, and plated for the analysis. Data are expressed as the ratio of 4‐OHT‐ versus ETOH‐treated samples and reported as mean (±SD). The *p*‐value was calculated by two‐sided Student's *t*‐test (*p < 0.05). (E) Neurosphere formation assay from P7 SmoA1/*Nbn* ^WT^ and SmoA1/*Nbn* ^HZ^ GCPs. Neurospheres were dissociated and plated for analysis in the presence of the indicated concentrations of SAG. Data are expressed as the ratio of SmoA1/*Nbn* ^HZ^ versus SmoA1/*Nbn* ^WT^ spheres for each concentration. *p*‐Values were calculated by two‐sided Student's *t*‐test (*p < 0.05, **p < 0.01, ***p < 0.001).

Despite the biochemical activation of a typical DDR, we did not observe reduction either in EdU incorporation nor reduction of the HH targets *Gli1* and *MycN* in surviving cells (Figure 3A,B,C). To our surprise, we found that S‐cNS with 4‐OHT‐induced *Nbn* hemizygosis was reproducibly able to re‐form more neurospheres compared to the control‐treated cells (Figure 3D), suggesting that *Nbn* hemizygosis may increase clonogenic ability in GCPs. By contrast, we did not observe similar results in 4‐OHT‐treated S‐cNS from *Nbn* ^F6/+^;Gli1CreER^−/−^ mice (Figure S5C), excluding that the clonogenic increase was directly due to 4‐OHT. Similar data were also obtained in the SmoA1‐dependent tumorigenic context (Figure 3E), where the differences between SmoA1/*Nbn* ^HZ^ and SmoA1/*Nbn* ^WT^ cultures were exacerbated by decreasing the concentration of SAG (Figure 3E), implicating that additional SHH‐independent activity may also contribute to clonogenesis in SmoA1/*Nbn* ^HZ^ S‐cNS compared to SmoA1/*Nbn* ^WT^ ones.

**FIGURE 4:**
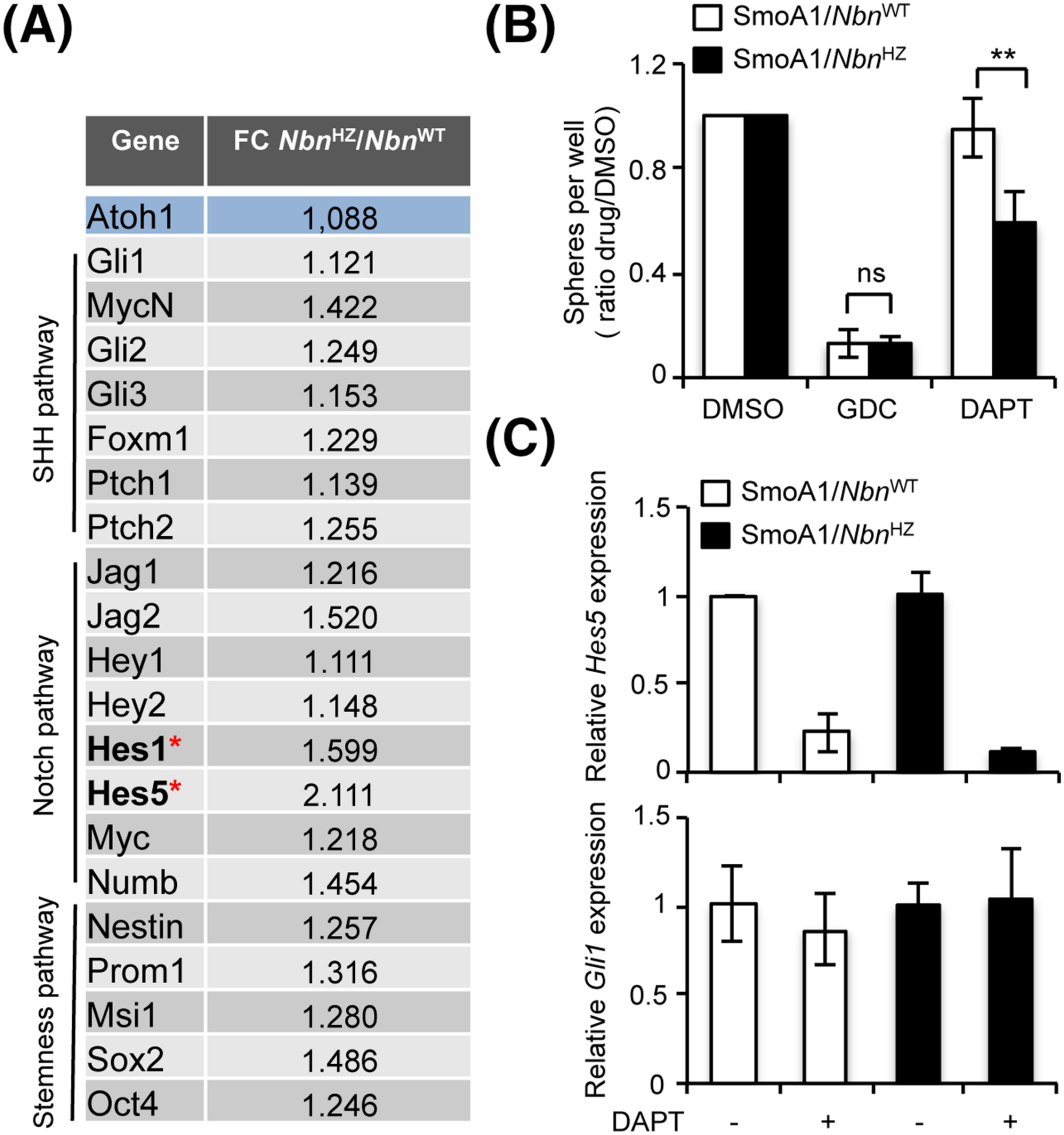
The enhanced clonogenicity of SmoA1/*Nbn* ^HZ^ granule cell progenitors (GCPs) depends on Notch signalling. (A) Microfluidic card qPCR analysis of the indicated transcripts in SmoA1/*Nbn* ^WT^ and SmoA1/*Nbn* ^HZ^ GCP neurospheres. mRNA expression levels were normalised on the mean expression of four reference genes (*Pgk1*, *Hprt*, *Gusb*, and *Tfrc*), and relative mRNA quantification was expressed as fold change. Data are reported as mean (±SD) of five replicates. *p*‐Values were calculated by two‐sided Student's *t*‐test. Statistically significant differences (*p* < 0.05) were observed for *Hes1* and *Hes5* (marked with a red asterisk). (B) Neurosphere formation assay from P7 SmoA1/*Nbn* ^WT^ and SmoA1/*Nbn* ^HZ^ GCPs. Neurospheres were dissociated and plated for the analysis in the presence of DMSO, DAPT or GDC‐0449. Data are expressed as the ratio of drug‐ versus DMSO‐treated samples. *p*‐Values were calculated by two‐sided Student's *t*‐test (**p < 0.01; ns, not significant). (**C**) qPCR analysis of the indicated transcripts in GCP neurospheres treated as in B

Notch and stemness pathways have been previously involved in GCP expansion [44, 45]. Interestingly, most of the representative transcripts for these pathways were slightly more expressed in SmoA1/*Nbn* ^HZ^ than SmoA1/*Nbn* ^WT^ S‐cNS (Figure 4A). For *Hes1* and *Hes5* transcripts, such differences reached statistical significance, suggesting a causal role of the Notch pathway in promoting clonogenesis in SmoA1/*Nbn* ^HZ^. Of interest, although the SHH inhibitor GDC‐0049 inhibited clonogenesis and *Gli1* expression in both SmoA1/*Nbn* ^HZ^ and SmoA1/*Nbn* ^WT^ S‐cNS (Figure 4B, S6A), confirming they are both strictly dependent on the SHH pathway, the Notch inhibitor DAPT impaired clonogenesis only in the SmoA1/*Nbn* ^HZ^ cultures (Figure 4B). Because DAPT inhibited Notch pathway in both S‐cNS cultures, as indicated by a similar decrease in *Hes5* expression (Figure 4C), the data suggest that the Notch pathway is essential for clonogenesis only in the SmoA1/*Nbn* ^HZ^ S‐cNS. Importantly, DAPT did not alter *Gli1* expression indicating that the HH pathway remained unaffected (Figure 4C). Similar results were also reproduced in *Nbn* ^F6/+^;Gli1CreER^+/−^ S‐cNS with a *Nbn* knockout acutely induced by 4‐OHT treatment (Figure S6B).

Collectively, these data indicate that *Nbn* hemizygosis increases clonogenic capability, at least partially through Notch signalling, in preneoplastic GCPs.

### ***Nbn*** **is essential for SHH‐driven MB development**

**FIGURE 5:**
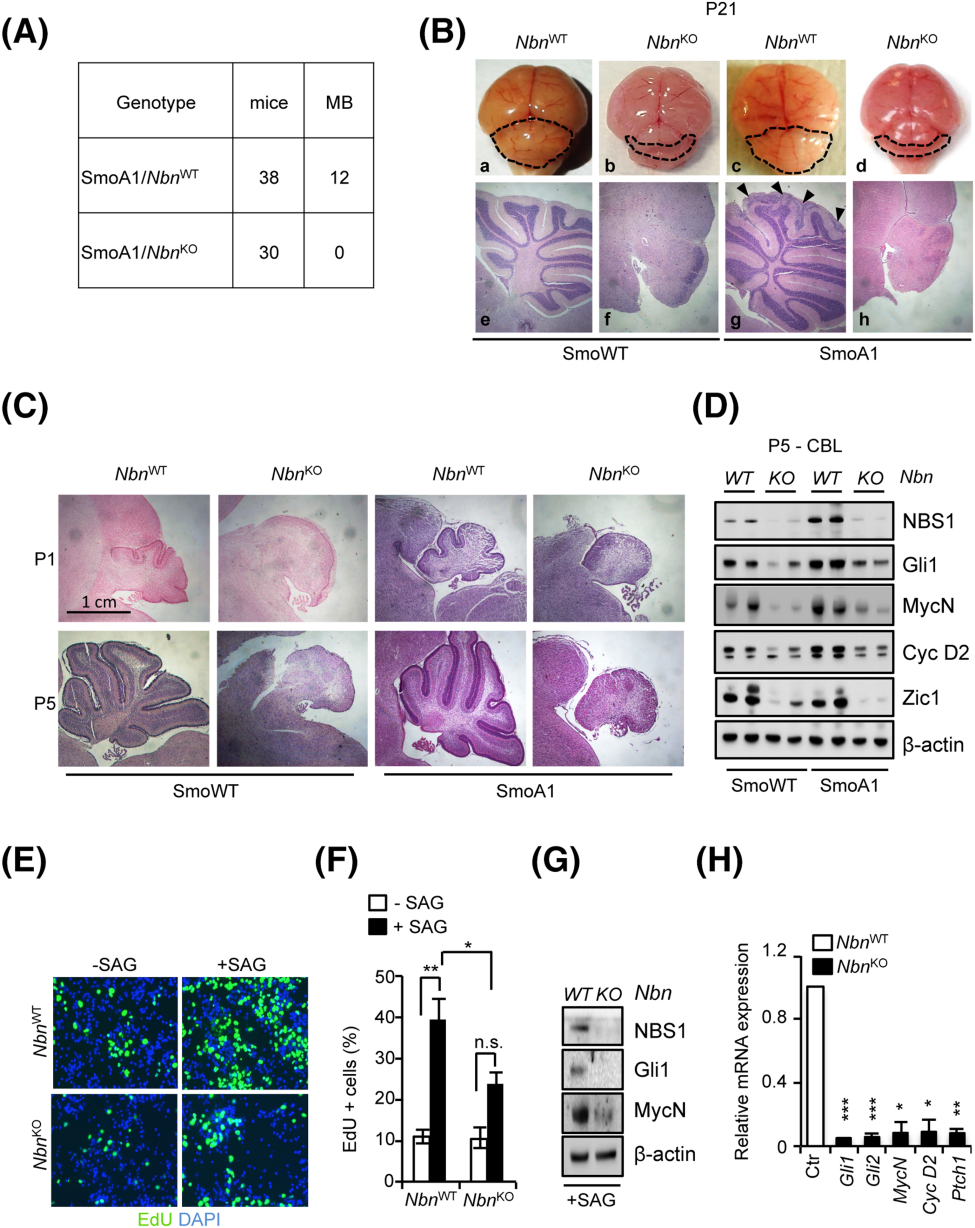
*Nbn* ^**KO**^ abolishes SmoA1‐dependent medulloblastoma (MB) and cerebellar development. (A) Summary of the animals developing MB in SmoA1/*Nbn* ^WT^ and SmoA1/*Nbn* ^KO^ mice. (B) Representative images of the macroscopic features (upper panel) and haematoxylin/eosin‐stained sagittal sections (lower panel) of the brain/cerebellum from P21 mice with the indicated genotypes. Dotted lines highlight the size/shape of the cerebella. Arrows in box g indicate regions of EGL hyperplasia. Original magnification (4×). (C) Representative images of haematoxylin/eosin‐stained sagittal sections of the P1/P5 cerebella explanted from mice with the indicated genotypes. Original magnification (4×). (D) Western blot (WB) analysis of protein extracts from P5 cerebella (n = 2). Blots were probed with the indicated antibodies, and β‐actin was used as a loading control. Data are representative of at least three replicates. **(**E, F) Representative images of EdU staining (E) and percentage of EdU‐positive cells (F) in primary GCPs were obtained from SmoWT/*Nbn* ^WT^ and SmoWT/*Nbn* ^KO^ cerebella, treated with or without SAG for 48 h. Data are reported as mean (±SD). *p*‐Values were calculated by ordinary one‐way ANOVA test. (G) WB analysis of primary granule cell progenitor (GCP) cultures generated from SmoWT/*Nbn* ^WT^ and SmoWT/*Nbn* ^KO^ cerebella. Data are representative of at least three replicates. (H) qPCR analysis of the indicated transcripts in primary GCP cultures. mRNA expression levels were normalised on two reference genes (*β2‐microglobulin* and *Hprt*), and relative mRNA quantification was expressed as fold change (FC). Data are reported as mean (±SD). *p*‐Values were calculated by two‐sided Student's *t*‐test (*p < 0.05, **p < 0.01, ***p < 0.001; ns, not significant).

Twelve out of 38 SmoA1/*Nbn* ^WT^ mice developed clinically evident MBs (Figure 5A,B). This was preceded by the occurrence of hyperplastic lesions in the EGL of fully developed cerebella (Figure 5B, box g), as described [34]. Coherently, enhanced SHH activity was revealed by the increased expression of its target genes Gli1, MycN and CycD2 and enlargement of the EGL during postnatal cerebellar development (Figure 5C,D). In sharp contrast, biallelic *Nbn* deletion resulted in complete suppression of SmoA1‐dependent EGL hyperplasia and MB development (Figure 5A,B). Rather, SmoA1/*Nbn* ^KO^ mice presented precocious signs of ataxia, growth retardation and a short lifespan, dying around weaning, substantially identical to the Smo^WT^/*Nbn* ^KO^ mice (Figure S7A‐C). Moreover, biallelic *Nbn* deletion resulted in impaired postnatal development of the cerebellum, which maintained an embryo‐like morphology with no fissures or folia formation, impaired expansion of the EGL, and persistently disorganised granule and Purkinje cell layers, all of which occurred equally in both WT and SmoA1 backgrounds (Figure 5C). This was accompanied by reduced cerebellar expression of SHH targets and of the GCP marker Zic1, in WT and SmoA1 backgrounds correspondingly (Figure 5D and S7D).

Importantly, we observed reduced SAG‐dependent proliferation and induction of SHH targets in primary GCPs isolated from P5 cerebella from *Nbn* ^KO^ compared to *Nbn* ^WT^ backgrounds (Figure 5E‐H). Consistently, we also observed impaired SHH‐dependent clonogenic growth of S‐cNS cultures from *Nbn* ^KO^ mice (Figure S7E).

Collectively, these results indicate that full *Nbn* ^KO^ impairs GCP postnatal expansion, preventing SmoA1‐driven hyperplasia and MB development. Because the phenotype of the SmoA1 model is driven by SHH activity, these results suggest that reduced proliferation and cell death driven by *Nbn* ^KO^ are epistatic on the SmoA1‐driven constitutive activation of the SHH pathway.

### ***Nbn*** **knockdown impairs SHH‐MB** **in** **vitro** **and** **in** **vivo**

**FIGURE 6:**
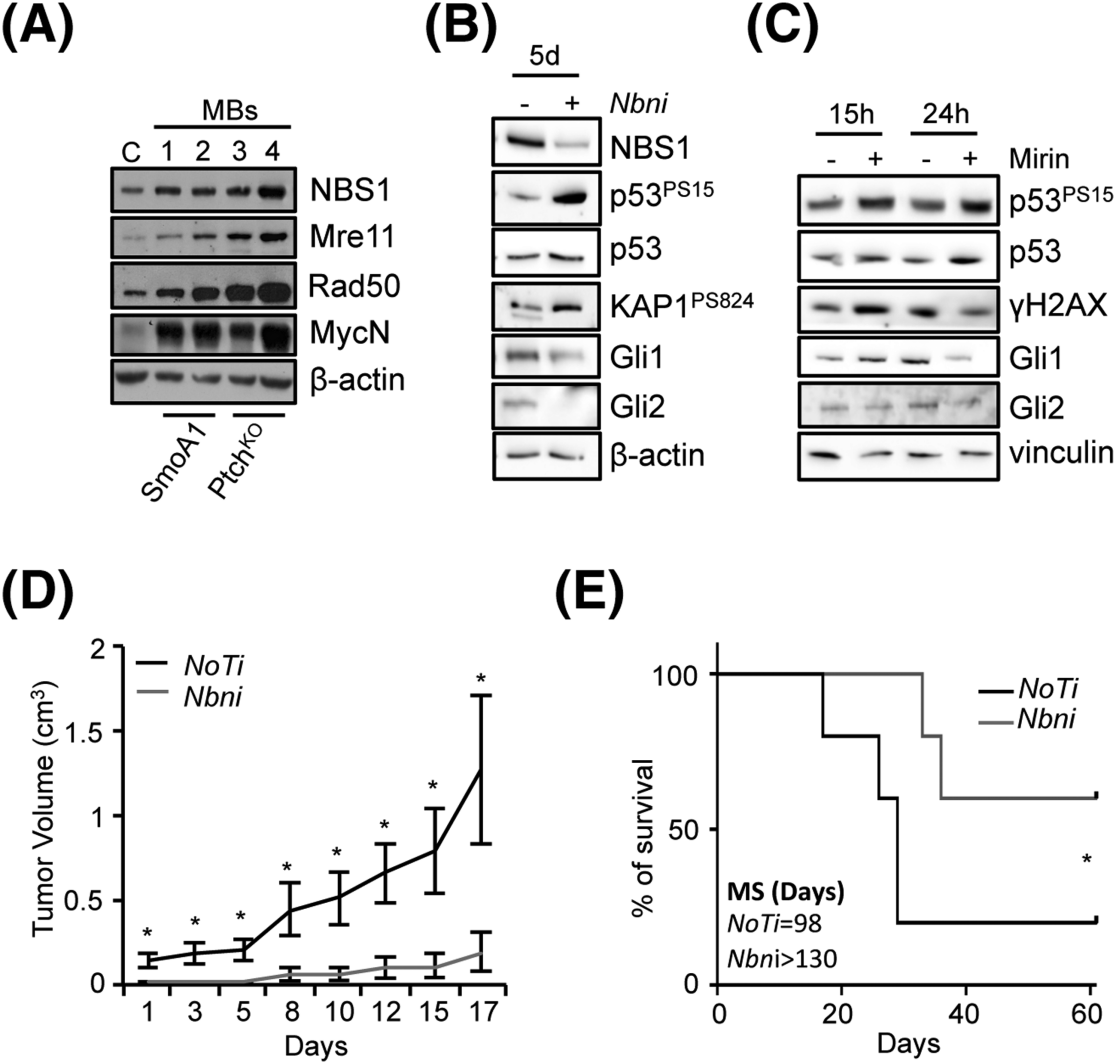
*Nbn* depletion impairs Sonic Hedgehog (SHH)‐medulloblastoma (MB) growth in vitro and in vivo. (A) Western blot (WB) analysis of healthy wild‐type (WT) cerebella at P21 (C) and MBs explanted from mice with the indicated genotypes. Blots were probed with the indicated antibodies, and β‐actin was used as a loading control. Data are representative of at least three replicates. (B) WB analysis of MB‐neurospheres subjected to *Nbn* RNAi (*Nbn*i) or no target RNAi (NoTi) for 5 days. (**C**) WB analysis of MB‐neurospheres treated with *mirin* or vehicle. Data are representative of three replicates. (D) evaluation of tumour growth in MB‐neurosphere allografts; data are expressed as mean volume ± SEM (n = 5 per group). Day 1 represents the first day in which tumour mass reached a measurable size. *p*‐Values were calculated by two‐sided Student's *t*‐test (*p < 0.05). (E) Kaplan–Meier survival curve of MB‐neurosphere allografts. Mice were sacrificed when tumours reached a volume of ≥2 cm^3^ or alternatively at Day 66. MS, median survival. *p*‐Values were calculated by a Gehan–Breslow–Wilcoxon test (*p < 0.05).

Although apparently counterintuitive, the idea that NBS1 is necessary for MB development is consistent with the previous data indicating that the MRN complex is required to control MYCN‐dependent RS and DNA damage in primary GCPs and in MYCN‐dependent tumours [18, 31, 46]. Consistent with this, we found higher MRN protein levels in SmoA1 and *Ptch1* ^KO^ MBs compared to adult (P21) WT cerebella (Figure 6A). Moreover, human SHH‐MBs had a significantly higher expression of MRN complex mRNA compared to normal cerebellar tissues (Figure S8), supporting the hypothesis that MRN functions might be essential in SHH‐MBs. To directly test this, we interfered *Nbn* gene expression in *Ptch* ^KO^ primary MB neurospheres. As expected, *Nbn* depletion induced DDR activation, indicated by the increase of p53 and KAP1 phosphoepitopes (Figure 6B). Notably, it also resulted in reduced expression of the HH targets Gli1 and Gli2 (Figure 6B). Similar data were obtained by treating MB neurospheres with the MRE11 inhibitor *mirin* (Figure 6C and S9A), which also led to cell proliferation arrest and cell death (Figure S9B, C). To test the dependency of SHH‐MB on MRN integrity in vivo, we allografted control and *Nbn*‐depleted primary MB neurospheres on the flank of nude mice (n = 5/condition) and monitored tumour growth. Remarkably, *Nbn* depletion slowed down tumour growth in vivo, allowing an increase in mice survival (Figure 6D,E).

These data indicate that *Nbn* is required for the growth of SHH‐MBs in vitro and in vivo and that its depletion affects the SHH pathway.

## DISCUSSION

**FIGURE 7:**
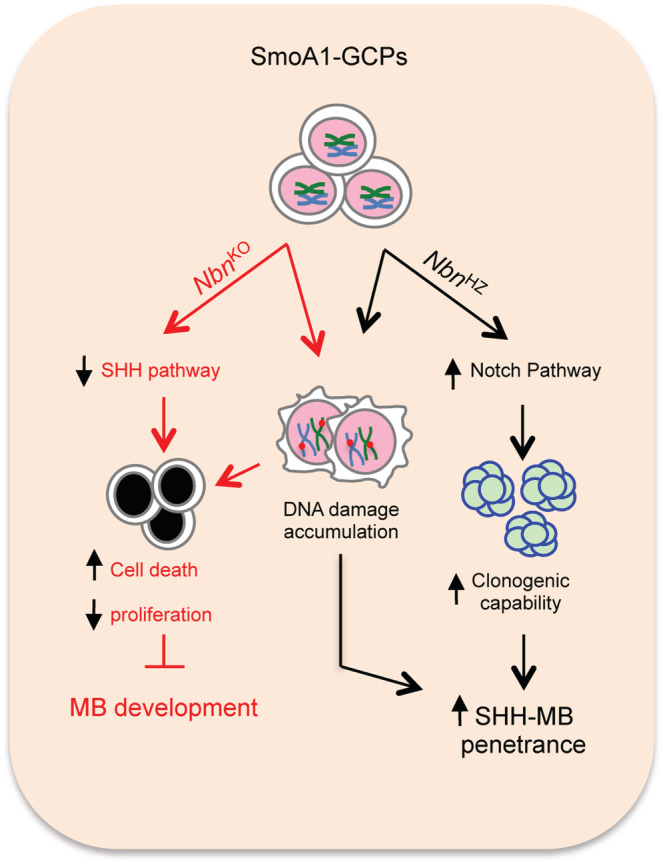
The Dual Role Of Nbs1 in Sonic Hedgehog (SHH)‐medulloblastoma (MB). In cancer prone SmoA1‐Granule Cell Progenitors (GCPs), *Nbn* ^KO^ causes reduced proliferation and increased cell death, ultimately leading to inhibition of MB development due to activation of the DNA damage response (DDR) and impairment of the SHH pathway. Whether these two events are linked or fully independent is currently not known. On the other hand, *Nbn* hemizygosis (*Nbn* ^HZ^) causes DDR activation and a Notch‐dependent increase in clonogenic capability, favouring GCP transformation. These results, collectively, indicate a gene dosage‐dependent function of *Nbn* in SHH‐driven tumorigenesis.

The MRN complex may exert both oncosuppressive and oncopromoting functions; however, as of today, this has not yet been clearly reconciled. Here, we report on a gene dosage‐dependent effect of *Nbn* ^KO^ on SHH‐MB development. In particular, we found that *Nbn* is haploinsufficient for SHH‐MB, for DDR and for the clonogenic growth of cerebellar GCPs, both of which emerge as relevant biological mechanisms involved in the development of SHH‐driven MB (Figure 7). In sharp contrast, full *Nbn* loss prevents both SHH‐MB and cerebellar development in a SmoA1‐dependent model (Figure 7).

The occurrence of MB in NBS patients and in heterozygous carriers and the presence of somatic *NBN* mutations in sporadic MB patients suggest that *NBN* might be haploinsufficient for MB development [27, 28, 29]. This has now been confirmed by our SmoA1/*Nbn* ^HZ^ model, where monoallelic *Nbn* ^KO^ significantly increases the likelihood of developing SHH‐MB in the absence of LOH. Rather, the reduced levels of NBS1 detectable in preneoplastic SmoA1/*Nbn* ^HZ^ cerebella appear to be compensated by an increased expression by the remaining allele in full‐blown MBs, which is in line with a widespread transcriptional activation of DNA repair and cell cycle genes in MB compared to healthy cerebella.

Full‐blown MBs appear morphologically identical in SmoA1/*Nbn* ^HZ^ and SmoA1/*Nbn* ^WT^ backgrounds and fail to show dramatic gene expression differences, perhaps suggesting that the mechanism/s enhancing cancer proneness in the former genotype operates at early stages of transformation. The increased level of chromosomal aberrations observed in *Nbn* ^HZ^ fibroblasts and tumour cells [22] and in *NBS1* and *RAD50* hypomorphic mutation carriers[42, 43, 47] suggests that the defective DDR in these individuals is the outcome of *Nbn* haploinsufficiency and the origin of genetic instability and cancer proneness. By showing that acute *Nbn* monoallelic KO induces H2AX and p53 phosphorylation and apoptosis in primary S‐cNS, we now add direct evidence that *Nbn* is haploinsufficient for the DDR‐associated replication‐born DNA damage in proliferating GCPs, the cells of origin of SHH‐MB. Much to our surprise, however, this did not lead to a reduction in EdU incorporation nor to the silencing of the SHH pathway in these cultures. Rather, *Nbn* ^HZ^ S‐cNS showed enhanced clonogenicity compared to *Nbn* ^WT^ cultures. This was further confirmed in SmoA1/*Nbn* ^HZ^ S‐cNS, where we also revealed upregulation of the Notch pathway compared to SmoA1/*Nbn* ^WT^ cultures. Consistently, the enhanced clonogenicity of SmoA1/*Nbn* ^HZ^ S‐cNS could be reduced by the Notch inhibitor DAPT. A role for the Notch pathway in SHH‐MB had been previously established by Hallahan et al, reporting increased expression of Notch targets in primary MBs and impaired growth/survival of SHH‐MB cells upon inhibition of Notch signalling [48]. Interestingly, Notch signalling appears to be mostly active in GCPs with more proliferative and immature properties, suggesting its expression is involved in regulating the proportion between GCPs and postmitotic GCs [49]. Our data, indicating the role of *Nbn* ^HZ^ on the enhanced and Notch‐dependent clonogenicity of GCPs, are also in line with the upregulation of the Notch pathway in lymphoblastoid cell lines from NBS heterozygous carriers as reported by Cheung *et al*. [50]. The emerging connection between NBS1 and the Notch pathway is further sustained by the observation that *Nbn* deletion elevates the expression of Notch targets via regulation of NICD‐RBPJ‐mediated transcriptional activity in a DDR‐independent manner [51]. Thus, both the Notch‐dependent enhanced clonogenicity and impaired DNA repair may be responsible for genetic instability and tumour proneness in *Nbn* ^HZ^ GCPs.

In sharp contrast, complete *Nbn* deletion fully prevents SmoA1‐dependent MB development due to an impairment in GCP proliferation and massive cell death, which are likely to overshadow other effects (i.e., on Notch signalling). Genetic or pharmacological inhibition of the MRN complex also impairs the growth of MB spheres in vitro and MB allografts in vivo, overall implying that MRN complex function/s are essential for SHH‐MBs. This is likely due to its role in preventing the deleterious accumulation of replication‐born DNA damage [52] exacerbated by MYCN‐induced replication stress in primary GCPs [18], which would result in a DDR and p53‐dependent cell cycle inhibition and/or cell death [16, 18, 52]. Consistently, p53^KO^ significantly rescues cerebellar defects in *Nbn* ^KO^ mice [16]. Of interest, the SmoA1/*Nbn* ^KO^ phenotype is essentially identical to the CNS‐*Nbn* ^KO^, suggesting that *Nbn* deletion is epistatic on constitutive SHH pathway activation because it not only impairs SmoA1‐dependent GCPs expansion in vivo but also inhibits SHH pathway and GCPs proliferation in a cell autonomous context in vitro. Due to the essential role of the SHH pathway for cerebellar development, our observations raise the hypothesis that the *Nbn* ^KO^‐dependent cerebellar defects and SHH‐MB prevention might also be linked to inhibition of the SHH pathway in addition to the well‐established link to DDR and p53 activation. It is worth recalling that p53 negatively controls Gli1 expression, its nuclear localization and activity, thus counteracting SHH signalling [45, 53, 54]. Moreover, CNS‐restricted deletion of diverse DNA repair genes systematically leads to SHH‐MBs in p53^−/−^ mice [6, 7, 8, 9], which might also be interpreted as the result of a specific function of p53 in shielding against SHH‐MB via repression of the SHH pathway. Notwithstanding these hypotheses, the molecular mechanism/s leading to the suppression of the SHH pathway in *Nbn* ^KO^ mice deserve further characterisation.

In conclusion, our work indicates that *Nbn* is haploinsufficient for SHH‐MB due to both DDR impairment and increased clonogenicity linked to the deregulation of the Notch pathway. Finally, biallelic *Nbn* ^KO^ appears epistatic on constitutive SHH pathway activation in GCPs, the molecular mechanisms of which needs to be further elucidated.

## CONFLICTS OF INTEREST

The authors declare that they have no known competing financial interests or personal relationships that could have appeared to influence the work reported in this paper.

## ETHICS STATEMENT

Animals were housed in cages with numbers and conditions according to the Italian ministry of Health and directive 2010/637EU guidelines. Animal experiments were approved by local ethics authorities (ministry autorization no. 379/2016‐PR).

## AUTHOR CONTRIBUTIONS

**MP, FF** and **SDG** performed the majority of the experiments, acquired and analysed the results, and wrote the manuscript. **GG** (corresponding author) conceived and supervised the project and wrote the manuscript. **VNdR, MM** and **SC** performed subcutaneous xenograft experiment and reviewed the manuscript. **VLM** and **FBu** assisted with all experiments and reviewed the manuscript. **FBe** performed Sanger sequencing and reviewed the manuscript. **PP** performed statistical and bioinformatic analyses and reviewed the manuscript. **ACor** performed histological analysis and reviewed the manuscript. **ACop**, **EDS**, **GC**, **LDM** and **ZQW** critically helped in project design, data analysis and manuscript writing.

### PEER REVIEW

The peer review history for this article is available at https://publons.com/publon/10.1111/nan.12837.

## Supporting information


**Table S1.** Primers sequences for PCR genotyping.
**Table S2.** Primers and probes sequences for qPCR genotyping.
**TableS 3.** List of qPCR assays used for mRNA analysis.
**Table S4.** List of CARD Assays used for mRNA analysis.
**Figure S1. *Generation of the* SmoA1*/Nbn*** ^**KO**^ ***and* SmoA1*/Nbn*** ^**HZ**^ ***mice models.***
**A,** Kaplan‐Meier survival curve of SmoA1 homozygous mice after repeated inter crossing. **B**, **C** PCR analyses **(B)** showing *Nbn* gene deletion and Western Blot (**C**) showing NBS1 protein down‐regulation in P1 cerebella. *Wt*: Wild type allele; *Floxed*: allele modified by flanking exon 6 with two loxP sites; *Δ6*: cleaved allele. β‐actin was used as loading control. WT: Wild type; HZ: mono‐allelic KO; KO: bi‐allelic KO. **D,** Western Blot analysis confirming the expression of SmoA1 transgene in the new mouse models by the Myc‐tag detection in P1 cerebella. Cerebella from SmoA1 and SmoWT mice were used as positive and negative controls for SmoA1 expression, respectively.
**Figure S2. *Global gene expression analysis of MBs and healthy cerebella in SmoA1/Nbn*** ^***WT***^ ***and SmoA1/Nbn*** ^***HZ***^ ***mice.***
**A**, Scatter plot of the RNA‐Seq expression data in SmoA1/*Nbn* ^WT^ MB versus healthy cerebella. Data are reported as the mean of the gene expression levels (in log‐scale) of three independent experiments. **B**, Scatter plot of the RNASeq expression data in SmoA1/*Nbn* ^HZ^ MB versus healthy cerebella. Data are reported as the mean of the gene expression levels (in log‐scale) of three independent experiments.
**Figure S3. A*nalysis of the DDR pathway in MBs originated from SmoA1/Nbn*** ^***WT***^ ***and SmoA1/Nbn*** ^***HZ***^ ***mice.***
**A**, WB analysis of MB (n=5) explanted from mice with the indicated genotypes. Blots were probed with the indicated antibody and β‐actin was used as loading control. **B**, **C**, **D**, **E**, **F**, heatmaps from RNA‐seq of the indicated transcripts in MBs and healthy adult cerebella (CBL; 15 months) of SmoA1/*Nbn* ^WT^ and SmoA1*/Nbn* ^HZ^ mice.
Figure S4. Analysis of the NBS1 expression in pre‐tumor and MBs originated from SmoA1/*Nbn* ^WT^ and SmoA1/*Nbn* ^HZ^ mice.
**A**, WB analysis of MBs (n=5) explanted from symptomatic mice with the indicated genotypes at around 7 months. Blot was probed with a Nbn antibody and β‐actin was used as loading control **B**, **C**, WB analysis of developing cerebella (n=6) explanted from mice with the indicated genotypes at P7 **(A)** and P14 **(B)**. Blots were probed with a Nbn antibody and β‐actin was used as loading control. *P7 N°4 sample was loaded in all three blots to allow a proper comparison of NBS1 expression levels in the different blots. **D,** Box Blot representing the median Nbn protein expression in the indicated samples. Densitometry was performed from WB in A, B and C using ImageJ software (version 2.0.0‐rc‐69/1.52n). p value was calculated by two‐sided Student's *t*‐test (*p < 0.05, **p < 0.01, n.s. not significant).
**Figure S5. *Generation and characterization of the Nbn*** ^***F6/+***^ ***;Gli1CreER*** ^***+/−***^ ***cell model.***
**A**, Schematic representation of the constructs used for the generation of the *Nbn* ^F6/+^;Gli1CreER^+/−^ mice. **B**, WB analysis of the *Nbn* ^F6/+^;Gli1CreER^−/−^ cells treated with ETOH or 4‐OHT for 24 hours and collected after 48 and 72 hours from the wash out. Blots were probed with the indicate antibodies and vinculin was used as loading control. **C**, Neurosphere formation assay from P7 *Nbn* ^F6/+^;Gli1CreER^−/−^ GCPs. Cultures were treated with ETOH or 4‐OHT for 24 hours, dissociated and plated for the analysis. Data are expressed as ratio of 4‐OHT versus ETOH treated samples and reported as mean (±SD).
Figure S6. *Involvement of the Notch pathway in the enhanced clonogenicity of the SmoA1/Nbn^HZ^ S‐cNS.*
**A,** Q‐PCR analysis of Gli1 mRNA expression in GCP neurospheres treated with GDC‐0449 for 24 hours. **B,** Neurosphere formation assay from P7 *Nbn* ^F6/+^; Gli1CreER^+/−^ GCPs. Neurospheres were treated with ETOH or 4‐OHT for 24 hours and then dissociated and plated for the analysis, in the presence of DMSO, DAPT or GDC‐0449. Data are expressed as ratio of 4‐OHT versus ETOH treated samples and reported as mean (±SD) of three independent experiments. P value was calculated by two‐sided Student's *t*‐test (*p < 0.05).
**Figure S7. *Additional features of SmoA1/Nbn*** ^***KO***^ ***and Smo/Nbn*** ^***KO***^ ***mice.***
**A**, Representative pictures of the mice with the indicated genotypes at P21. **B**, Relative weight analysis of mice with the indicated genotypes at P14 and P21. Data are reported as mean (±SE) of at least 6 independent biological replicates. The values for each condition are represented as FC with respect to the corresponding weights at P7. P value was calculated by two‐sided Student's *t*‐test (*p < 0.05, ***p < 0.001, ns: not significant). **C**, Survival analysis of the *Nbn* ^KO^ mice in the Smo^WT^ and SmoA1 backgrounds. Data are reported as mean (±SE) of at least 4 animals. **D**, qPCR analysis of the indicated transcripts in cerebella explanted from P5 mice with the indicated genotype. mRNA expression levels were normalized on the mean of expression of two reference genes (*β2‐microglobulin, Hprt*) and relative mRNA quantification was expressed as FC. Data are reported as mean (±SD). *P* values were calculated by two‐sided Student's *t*‐test (*p < 0.05, **p < 0.01, ***p < 0.001). **E**, Neurosphere formation assay from P5 *Nbn* ^WT^ and *Nbn* ^*K*O^ cerebellar explants grown with SAG. Data are expressed as ratio of *Nbn* ^KO^ versus *Nbn* ^WT^ samples and reported as mean (±SD). (**p < 0.01).
**Figure S8. Expression of MRN complex genes in human samples.** Box plots representing *MRE11, RAD50* and *NBN* mRNA expression in healthy cerebella and SHH‐MBs (Roth‐9‐MAS5.0 and SHH‐Pfister ‐73‐MAS5.0 datasets, respectively) obtained from the R2‐Genomics platform.
Figure S9. *Mirin impairs both SHH pathway and survival in MB‐neurospheres.*
**A**, qPCR analysis of the indicated transcripts in MB‐neurospheres treated with mirin or DMSO for 15 hours. mRNA expression levels were normalized on the mean of expression of two reference genes (*β2‐microglobulin, Hprt*) and relative mRNA quantification was expressed as FC. Data are reported as mean (±SD). P values were calculated by two‐sided Student's *t*‐test (**p < 0.01, ***p < 0.001). **B**, Representative images of EdU staining in MB‐neurospheres treated as in A. **C**, Representative images of alive (green) or dead (red) MB‐neurospheres treated with mirin or DMSO for 72 hours.

## Data Availability

The data that support the findings of this study are available from the corresponding author upon reasonable request.
