# Supplementary material for: A gene dosage‐dependent effect unveils NBS1 as both a haploinsufficient tumour suppressor and an essential gene for SHH‐medulloblastoma
Source: Neuropathol Appl Neurobiol. 2022 Aug 10;48(6):e12837. doi: 10.1111/nan.12837 (PMC9542137; doi:10.1111/nan.12837)
Supplement: Supplementary file 1 — Table S1. Primers sequences for PCR genotyping. Table S2. Primers and probes sequences for qPCR genotyping. TableS 3. List of qPCR assays used for mRNA analysis. Table S4. List of CARD Assays used for mRNA analysis. Figure S1. Generation of the SmoA1/Nbn KO and SmoA1/Nbn HZ mice models. A, Kaplan‐Meier survival curve of SmoA1 homozygous mice after repeated inter crossing. B, C PCR analyses (B) showing Nbn gene deletion and Western Blot (C) showing NBS1 protein down‐regulation in P1 cerebella. Wt: Wild type allele; Floxed: allele modified by flanking exon 6 with two loxP sites; Δ6: cleaved allele. β‐actin was used as loading control. WT: Wild type; HZ: mono‐allelic KO; KO: bi‐allelic KO. D, Western Blot analysis confirming the expression of SmoA1 transgene in the new mouse models by the Myc‐tag detection in P1 cerebella. Cerebella from SmoA1 and SmoWT mice were used as positive and negative controls for SmoA1 expression, respectively. Figure S2. Global gene expression analysis of MBs and healthy cerebella in SmoA1/Nbn WT and SmoA1/Nbn HZ mice. A, Scatter plot of the RNA‐Seq expression data in SmoA1/Nbn WT MB versus healthy cerebella. Data are reported as the mean of the gene expression levels (in log‐scale) of three independent experiments. B, Scatter plot of the RNASeq expression data in SmoA1/Nbn HZ MB versus healthy cerebella. Data are reported as the mean of the gene expression levels (in log‐scale) of three independent experiments. Figure S3. Analysis of the DDR pathway in MBs originated from SmoA1/Nbn WT and SmoA1/Nbn HZ mice. A, WB analysis of MB (n=5) explanted from mice with the indicated genotypes. Blots were probed with the indicated antibody and β‐actin was used as loading control. B, C, D, E, F, heatmaps from RNA‐seq of the indicated transcripts in MBs and healthy adult cerebella (CBL; 15 months) of SmoA1/Nbn WT and SmoA1/Nbn HZ mice. Figure S4. Analysis of the NBS1 expression in pre‐tumor and MBs originated from SmoA1/Nbn WT and SmoA1/Nbn HZ mice. A, WB [file NAN-48-0-s001.pdf]

A Gene dosage-dependent effect unveils NBS1 as both an haploinsufficient tumor suppressor and an essential gene for SHH-medulloblastoma

[1,6*]Marialaura Petroni, [1*]Francesca Fabretti, [1*]Stefano Di Giulio, [1]Vittoria Nicolis Di Robilant, [1]Veronica La Monica, [2]Marta Moretti, [1]Francesca Belardinilli, [1]Francesca Bufalieri, [2]Anna Coppa, [3,4]Paola Paci, [1]Alessandro Corsi, [2]Enrico De Smaele, [1]Sonia Coni, [1]Gianluca Canettieri, [1]Lucia Di Marcotullio, [5]Zhao-Qi Wang, [1,6]Giuseppe Giannini.

Supplementary Materials and Methods

Genomic DNA isolation from mouse tails

Mouse tissues were digested at 55 C° O/N in DNA extraction buffer (50mM Tris-HCl pH 8.0, 100mM NaCl, 100mM EDTA pH 8.0, 1% SDS) and Proteinase K (Sigma Aldrich); DNA was isolated by Phenol extraction technique and quantified by spectrophotometry (Nanodrop, ThermoFisher Scientific, Waltham, MA, USA). Approximately 100-200 ng of DNA was used for PCR analysis using Taq-Platinum polymerase (Invitrogen, Carlsbad, CA, USA). SmoA1 transgene copy number was calculated using qPCR analysis. Probes and primer sequences are given in Supplementary Tables 1 and 2.

GCPs adhesion medium

Neurobasal medium supplemented with 0.6% glucose, 25 μg/ml insulin (Sigma Aldrich), 60 μg/ml N-acetyl-L-cystein (Santa Cruz Biothecnology), 2 μg/ml heparin (Sigma Aldrich), penicillin-streptomycin, 2% B27 supplement without vitamin A (Life technologies), 2 μg/ml Heparin (Sigma Aldrich), 2 mM L-glutamine (Invitrogen) and SAG.

RNA sequencing

The quantification of transcripts was performed using HTSeq-count algorithm (Anders et al., Bioinformatics, 2015, https://doi.org/10.1093/bioinformatics/btu638). R was used to create a matrix of all transcripts expressed in all samples with the corresponding read-counts and the Bioconductor package DESeq247 was used to normalize the data and then to perform the differential expression analysis. DESeq2 package was used with standard parameters (Love et al., Genome Biol, 2014, https://doi.org/10.1186/s13059-014-0550-8).

Supplementary Table 1
Primer sequences for PCR genotyping.

Nbs1 exon 6 fw	5'-CAG GGC GAC ATG AAA GAA AAC-3'
Nbs1 LoxP rv	5'-AAT ACA GTG ACT CCT GGA GG-3'
Nbs1 intron 5 fw	5'-ATA AGA CAG TCA CCA CTG CG-3'
Cre fw	5'-CGG TCG ATG CAA CGA GTG ATG-3'
Cre rv	5'-CCA GAG ACG GAA ATC CAT CGC-3'
Norm fw	5'-CAC CGG AGA ATG GGA AGC CGA-3'
Norm rv	5'-TCC ACA CAG ATG GAG CGT CCA-3'

Supplementary Table 2
Primer and probe sequences for qPCR genotyping.

SmoA1 probe (FAM)	5'-CAGTGTCAGAACCCGCTGTTCACC-3'
SmoA1 Fw	5'-CAGACAACCCCAAGAGCTG-3'
SmoA1 Rv	5'-GTGCATGTCCTGGTGCTC-3'
Norm probe (VIC)	5'-CCAATGGTCGGGCACTGCTCAA-3'
Norm Fw	5'-CACGTGGGCTCCAGCATT-3'
Norm Rv	5'-TCACCAGTCATTTCTGCCTTTG-3'

Table 3

List of qPCR assays used for mRNA analysis.

Gli1: Mm00494654_m1
Gli2: Mm01293111_m1
MycN: Mm00476449_m1
Cyclin D2: Mm00438070_m1
Ptch1: Mm00436026_m1
Hes1: Mm01342805_m1
Hes5: Mm00439311_g1
B2M: Mm00437762_m1
HPRT: Mm00446966_m1

Table 4
List of CARD Assays used for mRNA analysis.

Atoh1: Mm00476035_s1
Gli1: Mm00494654_m1
MycN: Mm00476449_m1
Gli2: Mm01293117_m1
Gli3: Mm00492337_m1
Foxm1: Mm00514924_m1
Ptch1: Mm00436026_m1
Ptch2: Mm01187196_g1
Jag1: Mm00496902_m1
Jag2: Mm00439947_g1
Hey1: Mm00468865_m1
Hey2: Mm01180513_m1
Hes1: Mm01342805_m1
Hes5: Mm00439311_g1
Myc: Mm00487804_m1
Numb: Mm00477927_m1
Nestin: Mm01223405_g1
Prom1: Mm01211402_m1
Msi1: Mm01203522_m1
Sox2: Mm03053810_s1
Oct4 (Pou5f1): Mm03053917_g1
Pgk1: Mm00435617_m1
Hprt: Mm00446966_m1
Gusb: Mm00446954_g1
Tfrc: Mm00441941_m1

Table 5
List of WB antibodies.

anti-MycN #SC-53993 (Santa Cruz Biotechnology)
anti-vinculin #SC-73614 (Santa Cruz Biotechnology)
anti-Cyclin D1 #SC-717 (Santa Cruz Biotechnology)
anti-Myc #C3956 (Sigma Aldrich)
anti-β-actin #A5441 (Sigma Aldrich)
anti-NBS1 #NB110-57272 (Novus Biologicals),
anti-NBS1 #ab32074 (Abcam, Cambridge, UK)
anti-ZIC1 ab72694 (Abcam, Cambridge, UK)
anti-Mre11 #ab65131 (Abcam, Cambridge, UK)
anti Rad50 #ab119708 (Abcam, Cambridge, UK)
anti-Gli1 #L42B10 (Cell Signaling Technology)
anti-phospho-histone H2AX (ser 139) #2577 (Cell Signaling Technology)
anti-phospho-p53 (Ser 15) #9284 (Cell Signaling Technology)
anti-p53 (IC12) #2524(Cell Signaling Technology)
anti-PARP1 #9542 (Cell Signaling Technology)
anti-pKAP1(S824) #A300-767A (Bethyl laboratories, Montgomery, TX, USA)

Table 6
Primer sequences for *Nbn* sequencing.

NBS A Fw: CCACCCATTGATGAACCAGC
NBS A Rv: GGTGACTTTAACTGCCGACA
NBS B Fw: TGAATCTAAGAAACAGCCTCCAG
NBS B Rv: TCTTCGTCGTCTTCTGCCAT
NBS C Fw: CTGCACGTTGAAGACAGGAG
NBS Cbis Fw: GGTCTTTCCTGCACGTTGAA
NBS C Rv: TGGGAGTGTGAAATTATGAGCTG
NBS D Fw: AACAGTAAACTTCCCTGTAACCA
NBS Dbis Fw: GGTCTTTCCTGCACGTTGAA
NBS E Fw TTACCGCAAACAACTGGACG
NBS F Fw TACCCACCTTGTCATGTCGG

Supplementary Figures

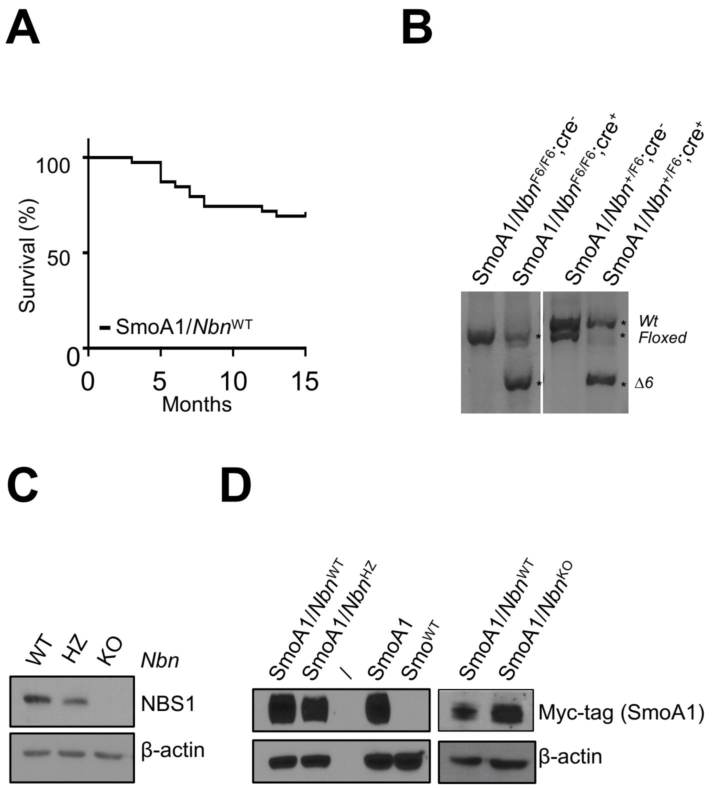

Petroni et al. Supplementary Figure 1.

Generation of the* SmoA1/*Nbn*^{KO} *and* SmoA1/*Nbn*^{HZ} *mice models.

A, Kaplan-Meier survival curve of SmoA1 homozygous mice after repeated inter crossing. **B**, **C** PCR analyses **(B)** showing *Nbn* gene deletion and Western Blot (**C**) showing NBS1 protein down-regulation in P1 cerebella. *Wt*: Wild type allele; *Floxed*: allele modified by flanking exon 6 with two loxP sites; *Δ6*: cleaved allele. β-actin was used as loading control. WT: Wild type; HZ: mono-allelic KO; KO: bi-allelic KO. **D,** Western Blot analysis confirming the expression of SmoA1 transgene in the new mouse models by the Myc-tag detection in P1 cerebella. Cerebella from SmoA1 and SmoWT mice were used as positive and negative controls for SmoA1 expression, respectively.

A

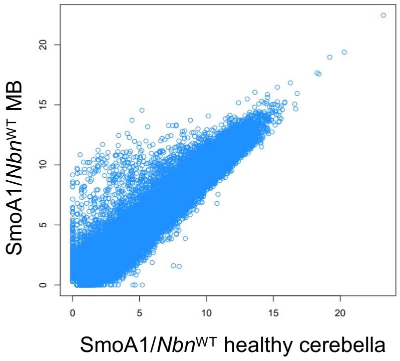

B

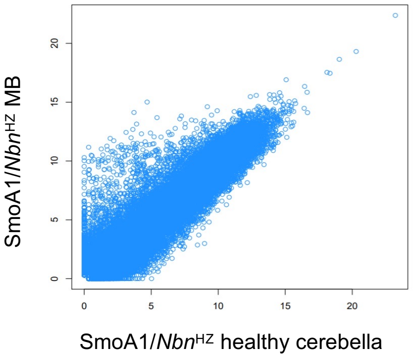

Petroni et al. Supplementary Figure 2

Global gene expression analysis of MBs and healthy cerebella in SmoA1/Nbn^{WT} and SmoA1/Nbn^{HZ} mice.

A, Scatter plot of the RNA-Seq expression data in SmoA1/*Nbn*^{WT} MB versus healthy cerebella. Data are reported as the mean of the gene expression levels (in log-scale) of three independent experiments·. **B**, Scatter plot of the RNA-Seq expression data in SmoA1/*Nbn*^{HZ} MB versus healthy cerebella. Data are reported as the mean of the gene expression levels (in log-scale) of three independent experiments**.**

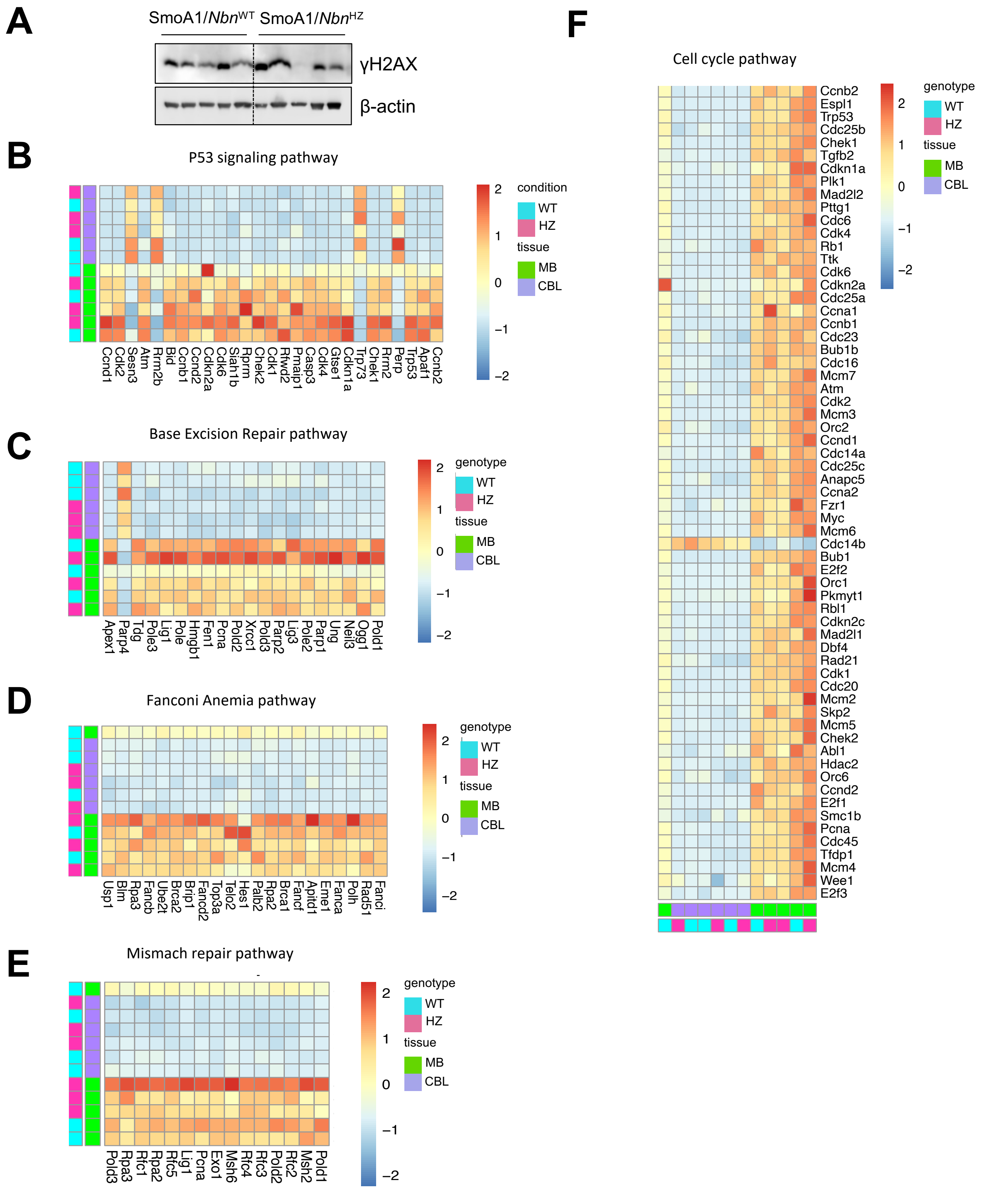

Petroni et al. Supplementary Figure 3

Analysis of the DDR pathway in MBs originated from SmoA1/Nbn^{WT} and SmoA1/Nbn^{HZ} mice.

A, WB analysis of MB (n=5) explanted from mice with the indicated genotypes. Blots were probed with the indicated antibody and β-actin was used as loading control. **B**, **C**, **D**, **E**, **F**, heatmaps from RNA-seq of the indicated transcripts in MBs and healthy adult cerebella (CBL; 15 months) of SmoA1/*Nbn*^{WT} and SmoA1/*Nbn*^{HZ} mice.

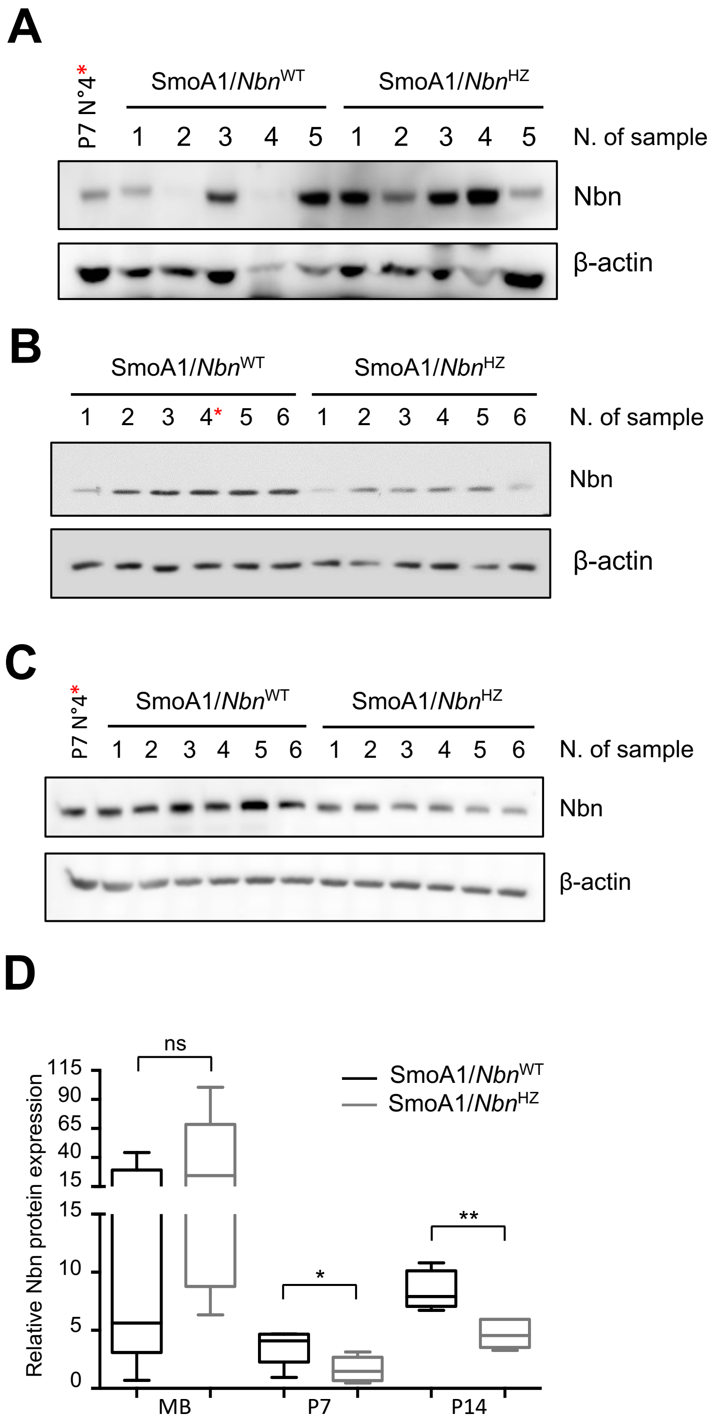

Petroni et al. Supplementary Figure 4

Analysis of the NBS1 expression in pre-tumor and MBs originated from SmoA1/Nbn^WT and SmoA1/Nbn^HZ mice.

A, WB analysis of MBs (n=5) explanted from symptomatic mice with the indicated genotypes at around 7 months. Blot was probed with a Nbn antibody and β-actin was used as loading control **B**, **C**, WB analysis of developing cerebella (n=6) explanted from mice with the indicated genotypes at P7 **(A)** and P14 **(B)**. Blots were probed with a Nbn antibody and β-actin was used as loading control. *P7 N°4 sample was loaded in all three blots to allow a proper comparison of NBS1 expression levels in the different blots. **D,** Box Blot representing the median Nbn protein expression in the indicated samples. Densitometry was performed from WB in A, B and C using ImageJ software (version 2.0.0-rc-69/1.52n). p value was calculated by two-sided Student's t-test ($*p < 0.05$, $**p < 0.01$, n.s. not significant).

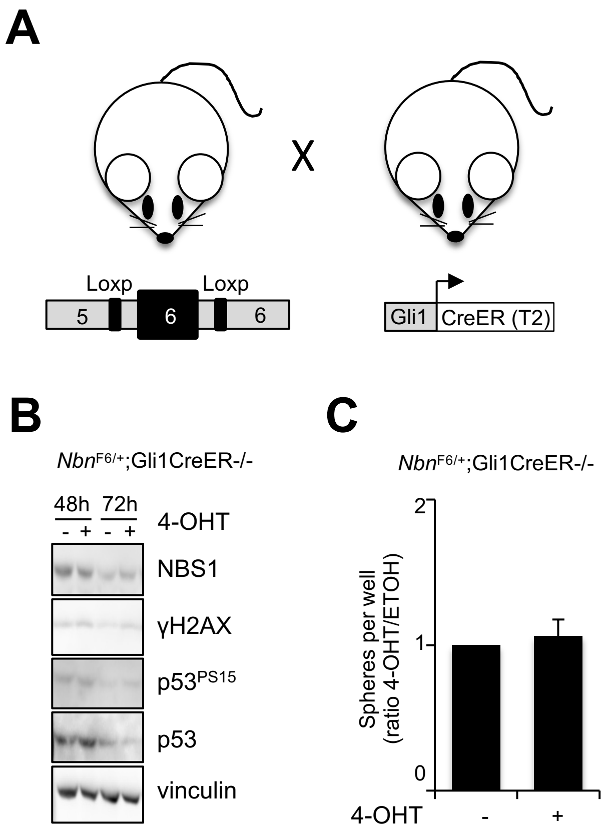

Petroni et al. Supplementary Figure 5

Generation and characterization of the Nbn$^{F6/+}$;Gli1CreER$^{+/-}$ cell model.

A, Schematic representation of the constructs used for the generation of the *Nbn*$^{F6/+}$;Gli1CreER$^{+/-}$ mice. **B**, WB analysis of the *Nbn*$^{F6/+}$;Gli1CreER$^{-/-}$ cells treated with ETOH or 4-OHT for 24 hours and collected after 48 and 72 hours from the wash out. Blots were probed with the indicate antibodies and vinculin was used as loading control. **C**, Neurosphere formation assay from P7 *Nbn*$^{F6/+}$;Gli1CreER$^{-/-}$ GCPs. Cultures were treated with ETOH or 4-OHT for 24 hours, dissociated and plated for the analysis. Data are expressed as ratio of 4-OHT versus ETOH treated samples and reported as mean (±SD).

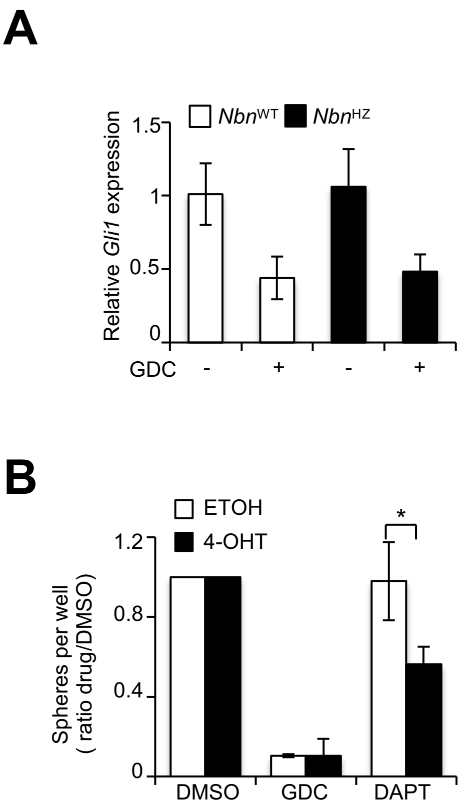

Petroni et al. Supplementary Figure 6

Involvement of the Notch pathway in the enhanced clonogenicity of the SmoA1/Nbn^{HZ} S-cNS.

A, qPCR analysis of Gli1 mRNA expression in GCP neurospheres treated with GDC-0449 for 24 hours. **B,** Neurosphere formation assay from P7 *Nbn*$^{F6/+}$;Gli1CreER$^{+/-}$ GCPs. Neurospheres were treated with ETOH or 4-OHT for 24 hours and then dissociated and plated for the analysis, in the presence of DMSO, DAPT or GDC-0449. Data are expressed as ratio of 4-OHT versus ETOH treated samples and reported as mean (±SD) of three independent experiments. p value was calculated by two-sided Student's t-test (*$p < 0.05$).

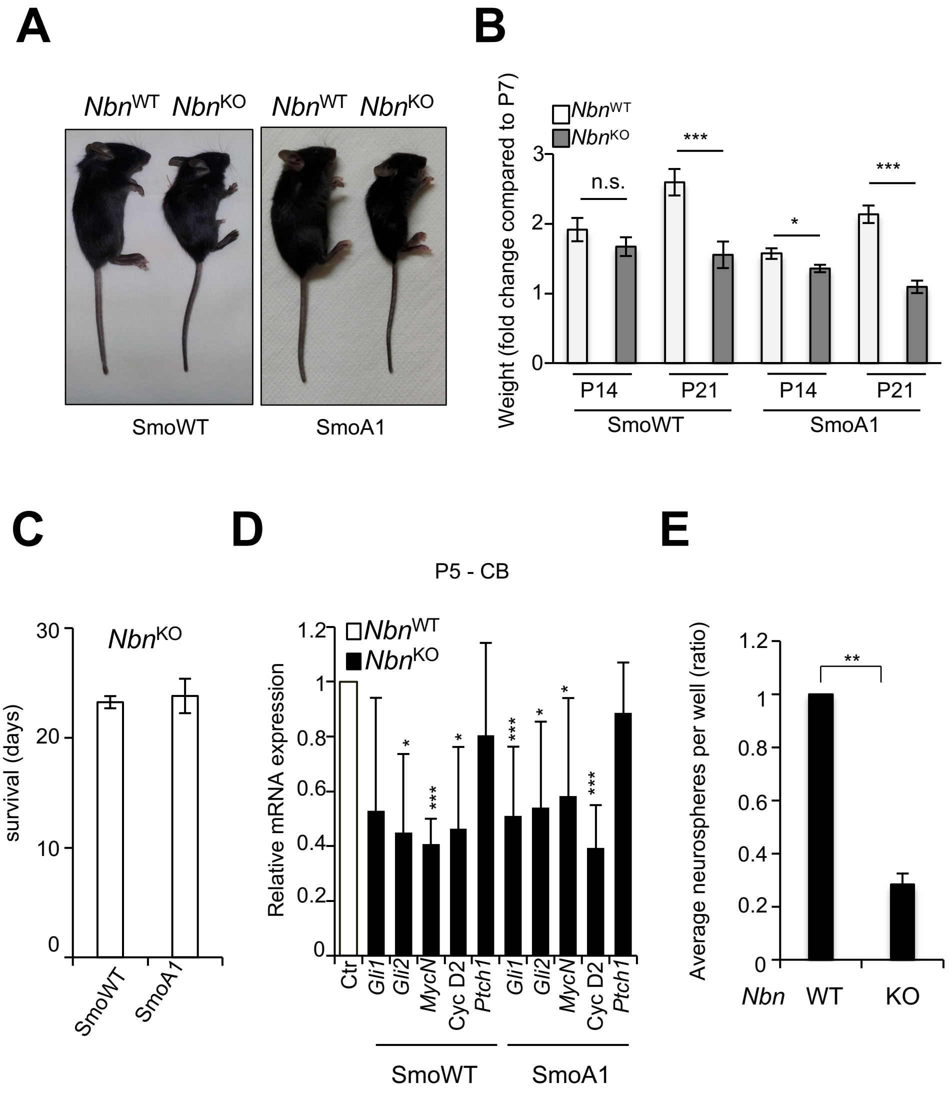

Petroni et al. Supplementary Figure 7

Additional features of SmoA1/Nbn^{KO} and SmoWT/Nbn^{KO} mice.

A, Representative pictures of the mice with the indicated genotypes at P21. **B**, Relative weight analysis of mice with the indicated genotypes at P14 and P21. Data are reported as mean (±SE) of at least 6 independent biological replicates. The values for each condition are represented as FC with respect to the corresponding weights at P7. p value was calculated by two-sided Student's t-test (*p < 0.05, ***p < 0.001, ns: not significant). **C**, Survival analysis of the *Nbn*^{KO} mice in the SmoWT and SmoA1 backgrounds. Data are reported as mean (±SE) of at least 4 animals. **D**, qPCR analysis of the indicated transcripts in cerebella explanted from P5 mice with the indicated genotype. mRNA expression levels were normalized on the mean of expression of two reference genes (*β2-microglobulin, Hprt*) and relative mRNA quantification was expressed as FC. Data are reported as mean (±SD). p values were calculated by two-sided Student's t-test (*p < 0.05, **p < 0.01, ***p < 0.001). **E**, Neurosphere formation assay from P5 *Nbn*^{WT} and *Nbn*^{KO} cerebellar explants grown with SAG. Data are expressed as ratio of *Nbn*^{KO} versus *Nbn*^{WT} samples and reported as mean (±SD). (**p < 0.01).

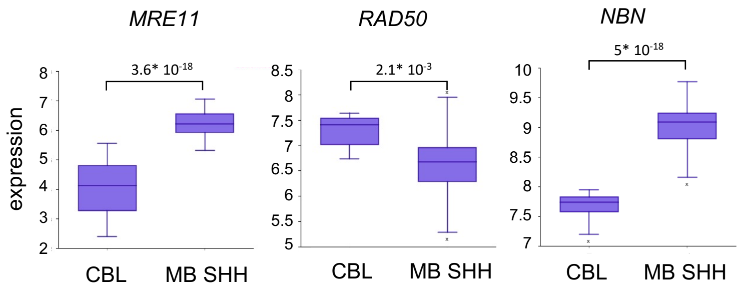

Petroni et al. Supplementary Figure 8

Expression of MRN complex genes in human samples. Box plots representing *MRE11, RAD50* and *NBN* mRNA expression in healthy cerebella and SHH-MBs (Roth-9-MAS5.0 and SHH-Pfister -73-MAS5.0 datasets, respectively) obtained from the R2-Genomics platform.

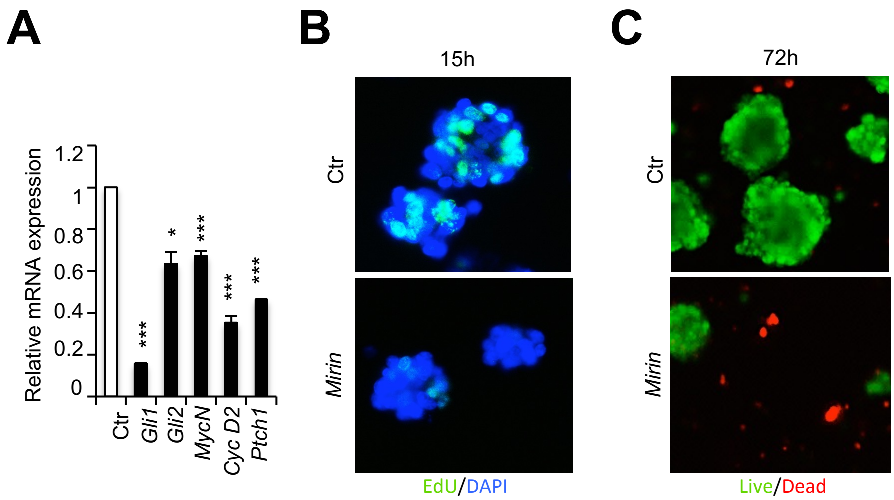

Petroni et al. Supplementary Figure 9

Mirin impairs both SHH pathway and survival in MB-neurospheres.

A, *q*PCR analysis of the indicated transcripts in MB-neurospheres treated with mirin or DMSO for 15 hours. mRNA expression levels were normalized on the mean of expression of two reference genes (*β2-microglobulin, Hprt*) and relative mRNA quantification was expressed as FC. Data are reported as mean (±SD). p values were calculated by two-sided Student's t-test (**$p < 0.01$, ***$p < 0.001$). **B**, Representative images of EdU staining in MB-neurospheres treated as in A. **C**, Representative images of alive (green) or dead (red) MB-neurospheres treated with mirin or DMSO for 72 hours.
